# Viral Replication Protein Inhibits Cellular Cofilin Actin Depolymerization Factor to Regulate the Actin Network and Promote Viral Replicase Assembly

**DOI:** 10.1371/journal.ppat.1005440

**Published:** 2016-02-10

**Authors:** Muhammad Shah Nawaz-ul-Rehman, K. Reddisiva Prasanth, Kai Xu, Zsuzsanna Sasvari, Nikolay Kovalev, Isabel Fernández de Castro Martín, Daniel Barajas, Cristina Risco, Peter D. Nagy

**Affiliations:** 1 Department of Plant Pathology, University of Kentucky, Lexington, Kentucky, United States of America; 2 Cell Structure Laboratory, Centro Nacional de Biotecnología (CNB-CSIC), Campus de Cantoblanco, Madrid, Spain; Agriculture and Agri-Food Canada, CANADA

## Abstract

RNA viruses exploit host cells by co-opting host factors and lipids and escaping host antiviral responses. Previous genome-wide screens with *Tomato bushy stunt virus* (TBSV) in the model host yeast have identified 18 cellular genes that are part of the actin network. In this paper, we show that the p33 viral replication factor interacts with the cellular cofilin (Cof1p), which is an actin depolymerization factor. Using temperature-sensitive (ts) Cof1p or actin (Act1p) mutants at a semi-permissive temperature, we find an increased level of TBSV RNA accumulation in yeast cells and elevated *in vitro* activity of the tombusvirus replicase. We show that the large p33 containing replication organelle-like structures are located in the close vicinity of actin patches in yeast cells or around actin cable hubs in infected plant cells. Therefore, the actin filaments could be involved in VRC assembly and the formation of large viral replication compartments containing many individual VRCs. Moreover, we show that the actin network affects the recruitment of viral and cellular components, including oxysterol binding proteins and VAP proteins to form membrane contact sites for efficient transfer of sterols to the sites of replication. Altogether, the emerging picture is that TBSV, via direct interaction between the p33 replication protein and Cof1p, controls cofilin activities to obstruct the dynamic actin network that leads to efficient subversion of cellular factors for pro-viral functions. In summary, the discovery that TBSV interacts with cellular cofilin and blocks the severing of existing filaments and the formation of new actin filaments in infected cells opens a new window to unravel the way by which viruses could subvert/co-opt cellular proteins and lipids. By regulating the functions of cofilin and the actin network, which are central nodes in cellular pathways, viruses could gain supremacy in subversion of cellular factors for pro-viral functions.

## Author Summary

The actin network, which is a central node in cellular pathways, is frequently targeted by various pathogens to modulate cellular responses. In this paper, the authors show that TBSV interacts with cofilin actin depolymerization factor leading to inhibition of the dynamic function of the actin network in infected cells. This allows TBSV to utilize the existing actin filaments to efficiently recruit host proteins and lipids for viral replication and to build viral replication compartments for robust viral replication. Altogether, subversion of the actin network by TBSV is a key step for the virus to gain access to cellular resources required for virus replication.

## Introduction

Plus-stranded (+)RNA viruses, which are important pathogens of plants, animals and humans, co-opt a number of host-coded proteins and lipids to facilitate the replication process [1–6]. These viruses also remodel host membranes and alter host cellular pathways to take advantage of host resources and to avoid recognition by host antiviral defenses. Characterization of an increasing number of host factors involved in (+)RNA virus replication has already revealed intriguing and complex interactions between various viruses and their hosts. Functional studies with selected host proteins have revealed a plethora of activities preformed by these host proteins during RNA virus infections [1,3,7–11]. In spite of the intensive efforts, our current cataloging of host factors is still far from complete and our current knowledge on the role of the identified host factors is incomplete.

One of the advanced viral systems to study virus-host interactions is *Tomato bushy stunt virus* (TBSV), a small (+)RNA virus, which can replicate in the model host yeast (*Saccharomyces cerevisiae*) [12–16]. TBSV replication requires two viral-coded proteins, namely the p33 and p92^pol^ replication proteins. Albeit these proteins have overlapping sequences, they have different functions. p33, which has RNA chaperone activity, has been shown to recruit the TBSV (+)RNA to the cytosolic surface of peroxisomal membranes, the sites of replication [17–20]. The p92^pol^ has RNA-dependent RNA polymerase (RdRp) activity and binds to p33 to assemble the membrane-bound functional viral replicase complex (VRC) [14,19,21–24].

The activities of TBSV replication proteins, however, are affected by numerous host proteins [3,7–9,25]. Indeed, over 500 host genes/proteins that affected TBSV replication and/or recombination, have already been identified by using multiple genome-wide screens of yeast and global proteomics approaches [16,26–31]. Moreover, the tombusvirus VRC contains several host proteins [32–34], including heat shock protein 70 (Hsp70), glyceraldehyde-3-phosphate dehydrogenase (GAPDH), eukaryotic elongation factor 1A (eEF1A), eEF1Bγ, the DDX3-like Ded1, eIF4AIII-like RH2 and DDX5-like RH5 DEAD-box RNA helicases, and the ESCRT (endosomal sorting complexes required for transport) family of host proteins [25,33,35–39]. These proteins are required for VRC assembly or affect viral RNA synthesis [3,35,38,40–42]. The TBSV replication process also depends on phospholipids and sterols, which are actively recruited to the site of viral replication [43–47].

Previous genomic and proteomic screens have revealed that Cof1p (cofilin in mammals) interacts with the tombusvirus p33 replication protein, and a mutation in *COF1* enhances TBSV RNA replication in yeast cells [30,48], suggesting that *COF1* could be an important host restriction factor. Cof1p is a major modulator of actin filament disassembly and an essential protein for yeast growth [49,50]. The major cellular function of Cof1p is to preferentially bind to ADP-actin subunits in actin filaments that results in twisting and severing the actin filaments [51,52]. Actin filament disassembly via Cof1p-induced depolymerization is required for remodeling of the actin cytoskeleton by providing free actin monomers as substrates for new filament formation [53–55]. Whether cofilin facilitates actin filament assembly or disassembly depends on the concentration of cofilin relative to actin. Overall, cofilins are conserved in eukaryotic cells and are essential from yeast to humans. Cofilins are known to affect many cellular pathways, including cell motility, cytokinesis, endocytosis, receptor functions, apoptosis, phospholipid metabolism, oxidative stress and gene expression via acting as a chaperone for nuclear actin [53,54,56,57]. Cofilins are also involved in several diseases, such as Alzheimer’s disease and ischemic kidney disease and other pathophysiological defects, such as infertility, immune deficiencies, inflammation, cancer, cognitive impairment [54,56,57].

Actin is highly conserved and abundant protein that exists in two forms in cells: globular monomeric (G-actin) and the active filamentous polymeric (F-actin) form. Actin undergoes multiple cycles of rapid nucleation, polymerization and disassembly, which is needed for remodeling the actin cytoskeleton. This organization of actin filaments is needed for vesicle transport, endocytosis, cell division and other functions in response to stimuli [58]. The cell frequently remodels the actin cytoskeleton with the help of ~100 highly conserved accessory proteins [58]. Regulation and activation of the accessory proteins, such as Arp2p and Arp3p actin nucleators and Cof1p, is needed to organize actin filaments in specific regions of the cytoplasm to carry out various functions.

Viruses dramatically reorganize the infected cells to support viral replication. Since the actin network is a key element in cell organization and transport of cellular proteins, lipids and intracellular organelles, viruses might target the actin network to reprogram cellular pathways and aid VRC assembly [59].

In this work, we show that cofilin actin depolymerization factor is targeted by tombusviruses to promote virus replication. Using temperature-sensitive (ts) Cof1p mutants at the semi-permissive temperature, we find increased level of TBSV RNA accumulation in yeast cells. The *in vitro* activity of the tombusvirus replicase prepared from *cof1-8* ^*ts*^ yeast was also higher than the activity of the replicase prepared from wt yeast. Also, over-expression of Cof1p or a plant homolog (Adf2) reduced TBSV accumulation in yeast. In addition, we show that TBSV replication depends on actin organization. Accordingly, inhibition of actin dynamic function via *act1* mutations in yeast or pharmacological inhibitors in plant cells led to increased TBSV replication. We demonstrate that the actin network affects the ability of TBSV to recruit host proteins and sterols to the viral replication sites. Altogether, the emerging picture is that TBSV, via direct interaction between p33 and Cof1p, controls cofilin activities to obstruct the dynamic actin network to lead to efficient subversion of cellular proteins and sterols for pro-viral functions.

## Results

### Cof1p is a regulator of TBSV replication

In our previous high throughput screens, we observed that the actin network was represented by one of the highest number of genes identified for TBSV (namely 18 genes). Particularly, we were interested in *COF1*, since Cof1p has been shown to interact with the tombusviral p33 replication protein [30]. Since Cof1p is a key actin depolymerization factor [54,55], binding of the tombusviral p33 replication protein to Cof1p might inhibit Cof1p interaction with ADP-actin and block actin disassembly and recycling of actin monomers to form new actin filaments.

**Fig 1:**
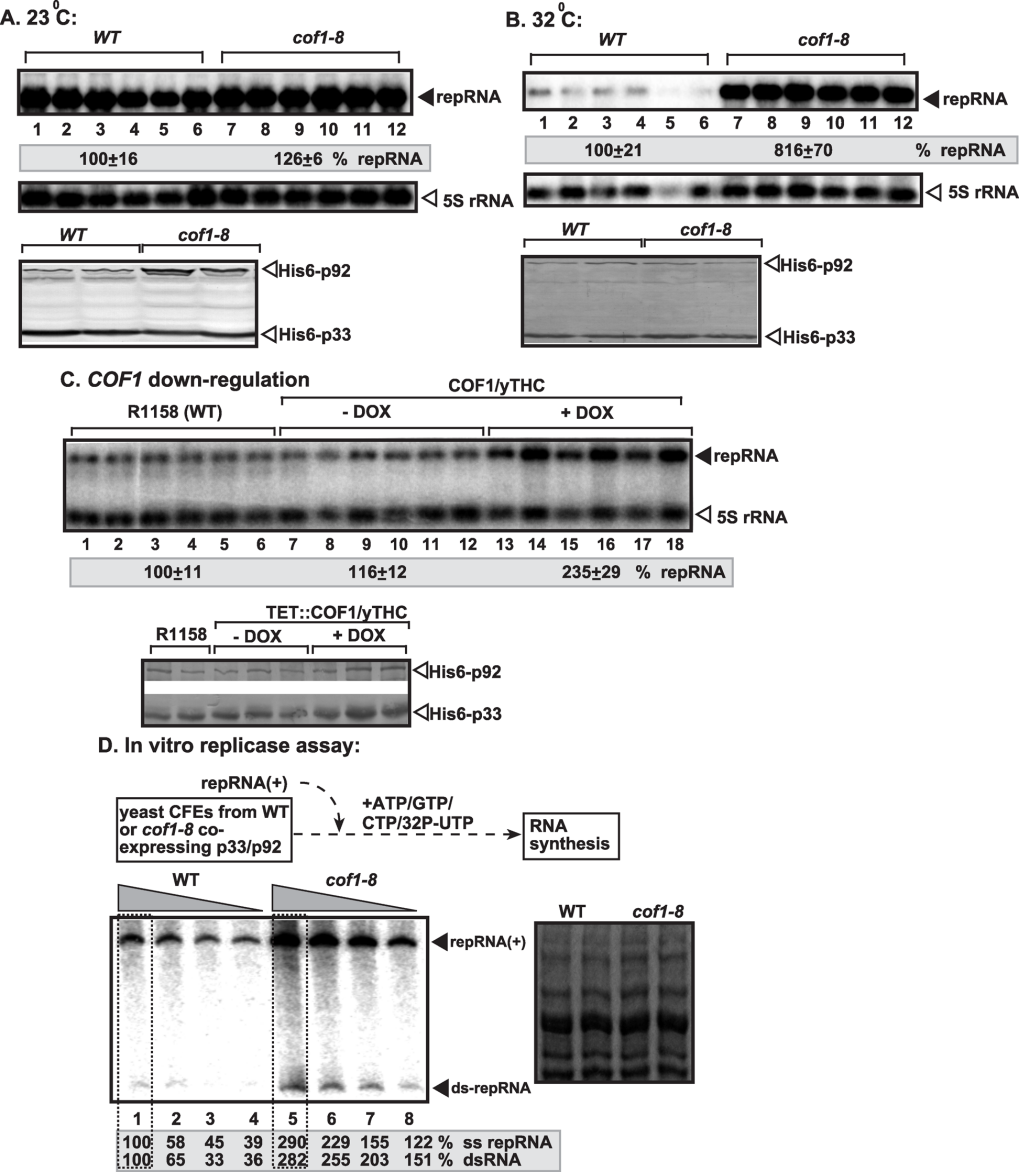
Cof1p inhibits TBSV repRNA accumulation in yeast. (A-B) Accumulation of TBSV repRNA in *cof1-8* ^*ts*^ or wt yeast at the permissive (23°C, panel A) or semi-permissive (32°C, panel B). To launch TBSV repRNA replication, we expressed His_6_-p33 and His_6_-p92 from the copper-inducible *CUP1* promoter and TBSV DI-72(+) repRNA from the constitutive *ADH1* promoter in the parental (BY4741) and *cof1-8* ^*ts*^ yeast strains. Northern blot analysis was used to detect DI-72(+) repRNA accumulation, which was normalized based on 5S rRNA. Typhoon FLA 9500 system (GE) and ImageQuant TL software were used to detect and quantify the bands in the gels. Each experiment was repeated three times. Bottom two panels: Western blot analysis of the accumulation level of His_6_-p33 and His_6_-p92 proteins using anti-His antibody. Throughout the paper, +/- means standard deviation. (C) Depletion of Cof1p level in yeast leads to increased repRNA accumulation. Note that doxycycline (+dox samples) leads to depletion of Cof1p expressed from the regulatable *TET* promoter. Replication of the TBSV DI-72(+) repRNA in wt and TET::COF1 yeasts co-expressing the tombusvirus p33 and p92 replication proteins was measured by Northern blotting 24 h after initiation of TBSV replication. Each sample is obtained from independent yeast colonies. The experiments were repeated two-to-three times. (D) Top: Scheme of the TBSV *in vitro* replication assay. The CFEs were prepared from wt or *cof1-8* ^*ts*^ yeast expressing the tombusvirus p33 and p92 replication proteins at the semi-permissive temperature. Then, the same amount (1 μg) of DI-72(+) repRNA was added to each reaction, followed by *in vitro* tombusvirus replication assay. Note that the CFE-based assay contained 4x (lanes 1 and 5), 3x (lanes 2 and 6) 2x (lanes 3 and 7) and 1x (lanes 4 and 8) amounts of CFEs. The non-denaturing PAGE analysis shows the emergence of single-stranded (+)repRNAs and dsRNAs (replication intermediate) as indicated. The image on the right shows the coomassie blue-stained total proteins in CFE preparations after SDS-PAGE analysis to show the presence of comparable amounts of total proteins in the CFEs.

To test if the interaction between Cof1p and p33 replication protein is relevant during TBSV replication, we used a temperature-sensitive (ts) Cof1p mutant yeast in viral replication studies. Interestingly, the data from Northern blot analysis showed that *cof1-8* ^*ts*^ yeast supported TBSV replicon (rep)RNA accumulation at ~8-fold increased level at the semi-permissive temperature in comparison with yeast carrying wt *COF1*, while the two strains showed comparable TBSV repRNA accumulation at the permissive temperature (compare lanes 7–12 versus 1–6, Fig 1A and 1B). These data indicate that Cof1p is an inhibitor of TBSV RNA replication in yeast host. The amount of p33 and p92^pol^ was comparable in these yeast strains at the semi-permissive temperature, suggesting that Cof1p does not affect translation or stability of the viral replication proteins. Using the *cof1-5* ^*ts*^ mutant of Cof1p also showed ~3.5-fold increased TBSV repRNA accumulation at the semi-permissive temperatures (S1A Fig), further supporting that Cof1p affects TBSV replication.

To obtain additional evidence on the inhibitory role of Cof1p, we down-regulated Cof1p level in yeast using the titratable *TET* promoter [60]. We found that TBSV repRNA accumulation increased by ~2.5-fold when Cof1p was down-regulated (lanes 13–18, Fig 1C). Thus, TBSV replication is stimulated by reduced level of Cof1p in yeast.

To test if Cof1p affects the viral replicase activity, we obtained cell-free extracts (CFE) from *cof1-8* ^*ts*^ and wt yeast, co-expressing p33 and p92^pol^. The CFE was programmed with the TBSV (+)repRNA *in vitro*, allowing one cycle of full TBSV repRNA replication [21]. We observed ~3-to-4-fold higher repRNA replication in CFE from *cof1-8* ^*ts*^ than in CFE from wt yeast (Fig 1D, lanes 5–8 versus 1–4). Interestingly, both the newly made (+)repRNA and double-stranded dsRNA (correlating with the minus-stranded RNA [61]) levels increased to a similar extent in CFE from *cof1-8* ^*ts*^ (Fig 1D). Thus, Cof1p affects the early stage of TBSV replication, possibly VRC assembly prior to (-)-strand RNA synthesis. Altogether, the *in vitro* data are consistent with the results from yeast, supporting that Cof1p is a negative regulator of TBSV replication.

### Over-expression of Cof1p inhibits TBSV RNA accumulation

**Fig 2:**
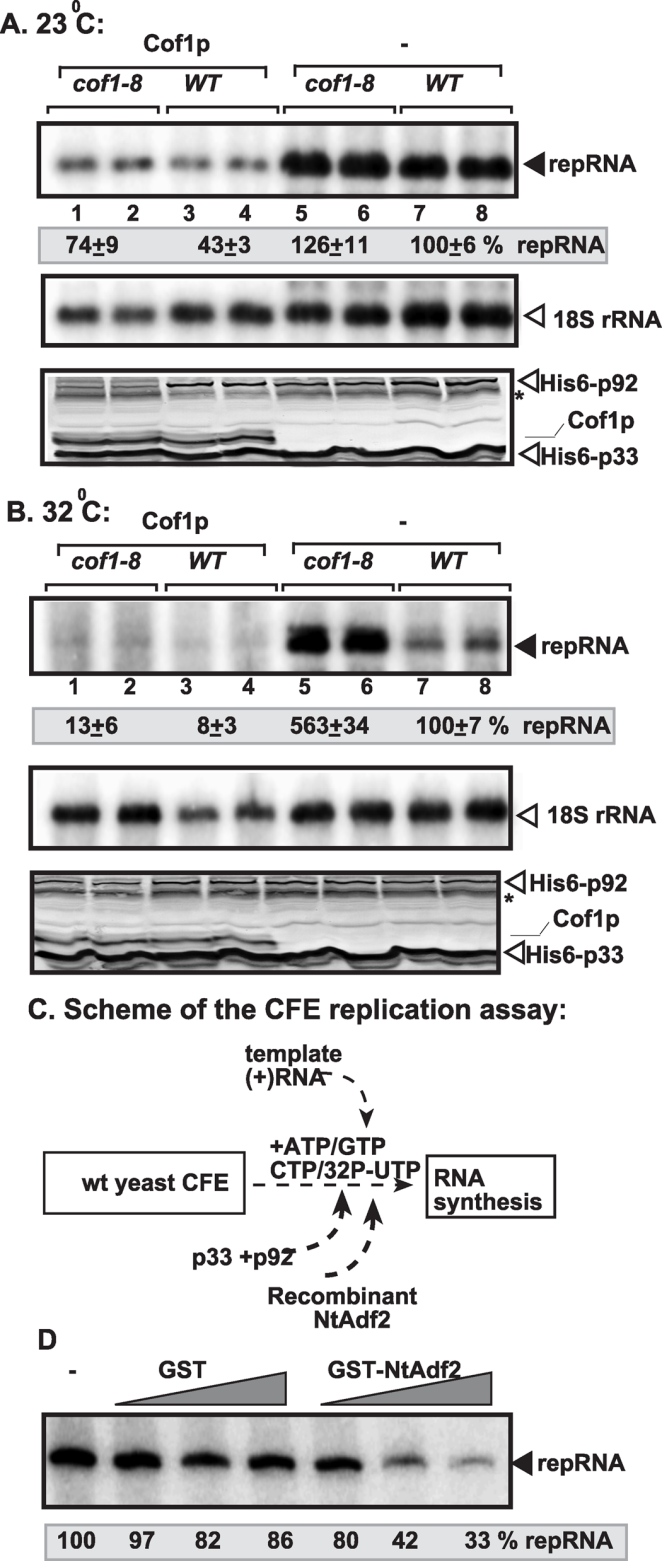
Over-expression of Cof1p inhibits TBSV repRNA accumulation in yeast. (A-B) The wt or *cof1-8* ^*ts*^ yeast co-expressed the His_6_-p33 and His_6_-p92 from the copper-inducible *CUP1* promoter and TBSV DI-72(+) repRNA from the constitutive *ADH1* promoter. Over-expression of His_6_-Cof1p was done from the *GAL1* promoter. Top images: Northern blot analysis of TBSV repRNA in yeast samples over-expressing Cof1p or without over-expression is shown. repRNA replication took place for 24 hours at 23°C or 32°C in wt or *cof1-8* ^*ts*^ yeast before RNA analysis. The accumulation level of DI-72(+) repRNA (shown in percentage) was normalized based on 18S rRNA. Middle panel shows the level of 18S rRNA. Bottom panels: Western blot analysis of the co-expressed 6xHis-tagged proteins with anti-His antibody is shown. (C) Scheme of the CFE-based TBSV replication assay. Purified recombinant p33 and p92^pol^ replication proteins of TBSV and *in vitro* transcribed TBSV DI-72 (+)repRNA were added to the CFE prepared from wt yeast strain. The affinity-purified recombinant *Nicotiana tabacum* Adf2 (Cof1 homolog) was added before the CFE-based replication assay as shown. (D) Inhibition of TBSV replication by recombinant NtAdf2 *in vitro*. Denaturing PAGE analysis of the ^32^P-labeled TBSV repRNA products obtained in the CFE-based TBSV replication assay in the presence of recombinant GST-NtAdf2p (1x = 200 ng) as shown. Each experiment was repeated three times.

Over-expression of the zz-His_6_-tagged Cof1p reduced TBSV repRNA accumulation by ~50% in both *cof1-8* ^*ts*^ and wt yeast at the permissive temperature (23°C, Fig 2A, lanes 1–4 and 5–8). The inhibitory effect of Cof1p was even more pronounced at the semi-permissive temperature (32°C), resulting in ~30-fold reduction in TBSV repRNA level in *cof1-8* ^*ts*^ yeast over-expressing Cof1p (Fig 2B, lanes 1–2 versus 5–6). The amount of p33 and p92^pol^ was comparable in these yeast strains at both temperatures. We also found a strong inhibitory effect on TBSV repRNA accumulation when the native (nontagged) Cof1p was over-expressed in *cof1-8* ^*ts*^ and wt yeast strains (S2 Fig).

To test if cofilin can directly inhibit TBSV replication, we purified recombinant Adf2 of *Nicotiana tabacum*, a plant homolog of the yeast Cof1p [62–64]. Adding purified recombinant NtAdf2 to yeast CFE (as shown schematically in Fig 2C) performing one full cycle of TBSV replication (including both minus- and plus-strand synthesis) resulted in up to 70% inhibition of TBSV RNA synthesis (Fig 2D). This experiment showed that cofilin/ADF could directly inhibit TBSV replication *in vitro*, likely due to sequestering the p33 replication protein.

### Formation of large tombusvirus replication compartments in cof1-8^ts^ yeast

**Fig 3:**
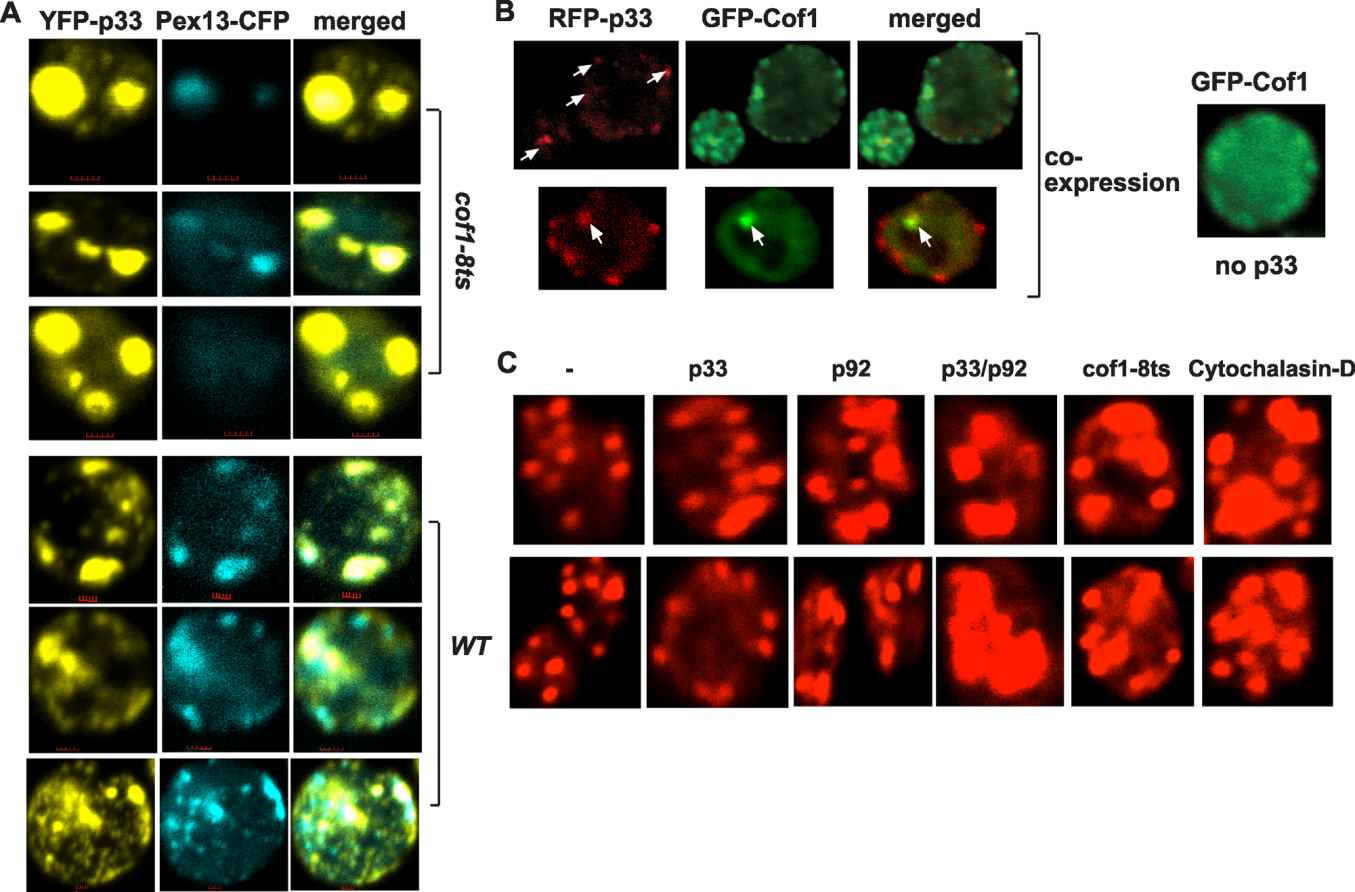
The viral replication proteins inhibit the actin network via interaction with Cof1p. (A) Confocal laser microscopy images show the co-localization of Pex13-CFP with YFP-p33 expressed from *ADH1* promoter in *cof1-8* ^*ts*^ or in BY4741 (parental strain) yeast strains at the semi-permissive temperature (32°C). The merged images show in single yeast cells the co-localization of Pex13-CFP with YFP-p33 in fewer but larger punctate structures, which is indicative of large replication organelles, in *cof1-8* ^*ts*^ than in wt yeast cells. Note that Pex13 represents a peroxisomal marker, where TBSV forms the VRCs and performs RNA replication. The bars represent 1 μm. (B) Partial co-localization of Cof1p and p33 replication protein in yeast cells. Top panel shows the early time point (5 h), while the bottom panel shows the late time point (24 h) after the induction of protein expression from the *GAL1* promoter in cof1-8^ts^ yeast cells. The shift to the semi-permissive temperature (32°C) was done 2 h prior to the induction of Cof1p and p33 expression. Note also the altered localization pattern of GFP-Cof1 in yeast co-expressing p33 in comparison with the more even distribution of GFP-Cof1 in the absence of viral components. The GFP-cofilin construct has S65T-GFP and a 12–amino acid linker inserted between amino acids N74 and G75 of Cof1p. (C) Rhodamine-phalloidin staining of actin in wt yeast expressing p33, p92, or p33+p92 replication proteins in comparison with *cof1-8* ^*ts*^ yeast (grown at the semi-permissive 32°C) or wt yeast treated with Cytochalasin-D, an inhibitor of new actin filament formation in yeast. Note that the control (left panels), *cof1-8* ^*ts*^ yeast and Cytochalasin-D treated yeasts do not have viral components. The larger size of cortical actin patches are due to the inhibition of actin monomer recycling and lack of dynamic new actin filament formation. Two sets of independent yeast cells are shown in each experiment.

To test if the higher level of TBSV RNA replication and the more active tombusvirus replicase from *cof1-8* ^*ts*^ yeast are due to the different subcellular localization or an altered distribution of the replication proteins, we used confocal laser microscopy imaging of live yeast cells. This study revealed that p33 replication protein formed a few large punctate structures in *cof1-8* ^*ts*^ yeast at the semi-permissive temperature in contrast with the more numerous, but smaller punctate structures in wt yeast (Fig 3). However, YFP-p33 was co-localized with the peroxisomal marker protein (Pex13p) in *cof1-8* ^*ts*^ yeast (Fig 3A), suggesting that p33 is localized to peroxisome-derived membranes in both *cof1-8* ^*ts*^ and wt yeast cells. Altogether, the presence of large replication foci in *cof1-8* ^*ts*^ yeast suggests that tombusvirus replication proteins are likely more efficient in organizing the viral replication compartments containing many VRCs than in wt yeast cells. The formation of large p33-containing peroxisomal structures in *cof1-8* ^*ts*^ yeast expressing the Cof1 ts mutant (Fig 3A) were similar to those observed with phospholipid synthesis mutants that also supported TBSV replication at a high level [44,46,65]. These data suggest efficient assembly of VRCs when cofilin is mutated.

To study if the presence of p33 changes the subcellular localization of Cof1p, we co-expressed RFP-tagged p33 and GFP-tagged Cof1p in *cof1-8* ^*ts*^ yeast cells at the semi-permissive temperature, followed by confocal laser microscopy. Interestingly, in the presence of p33, Cof1p showed not only diffused localization in the cytosol, but also formed several punctate structures, which frequently co-localized with p33 (Fig 3B, left images). In contrast, GFP-Cof1p was present mostly in the diffused form in the cytosol in *cof1-8* ^*ts*^ yeast in the absence of p33 (Fig 3B, right panel). These results indicate that a fraction of Cof1p is co-localized with p33 at both early (5 hour) and late time points, indicating that the co-expressed p33 can change the subcellular localization of Cof1p in *cof1-8* ^*ts*^ yeast. The viral replication protein-induced relocalization of Cof1p might inhibit the normal cellular function of Cof1p (see Discussion).

Since Cof1p is a key actin depolymerization factor [54,55], and binds to the tombusviral p33, it is plausible that p33 might inhibit Cof1p interaction with ADP-actin and block actin filament disassembly and recycling of actin monomers that is needed to form new actin filaments. Indeed, co-expression of p33 and p92 replication proteins in yeast led to the formation of a large actin-filament network (called actin patches, which contain short, branched actin filaments with 1–2 minute turnover time) in the cell, visible under confocal microscope (Fig 3C). Formation of large cortical actin patches is indicative of lack of Cof1-driven disassembly of actin filaments and inhibition of dynamic actin function [52,58]. Indeed, blocking Cof1p function via applying semi-permissive temperature in *cof1-8* ^*ts*^ yeast or using the actin polymerization inhibitor, Cytochalasin-D, has led to similar actin organization (large cortical actin patches) to that induced by p33/p92 expression (Fig 3C).

The cortical actin patches were also influenced by expressing either p33 or p92 alone, albeit the effects were lesser than those observed with co-expression of p33 and p92 (Fig 3C). These observations suggest that p33 and p92 interferes with the normal organization of actin in yeast, perturbing actin organization as efficiently as Cof1p mutation or chemical inhibition of actin polymerization. It is worthwhile to mention that p92 shares an identical N-terminal sequence with the full-length p33 and both p33 and p92 can affect actin organization under the experimental conditions (Fig 3C). Based on these observations, we propose that TBSV replication proteins actively block actin organization via the interaction of p33/p92 and Cof1p.

### Actin-organization function of Cof1p is needed for inhibition of tombusvirus replication

The best-characterized function of the multifunctional Cof1p is to bind to ADP-bound actin and depolymerize actin filaments in yeast cells [54,55]. To test if the actin-organization function of Cof1p is important for the inhibitory function of Cof1p on TBSV replication, we used a group of Cof1p mutants in the yeast-based TBSV replication assay. Interestingly, a Cof1p mutant that binds poorly to G- or F-actin (*cof1-22*) [66] supported TBSV replication at ~3-fold higher level than wt (S1B Fig). Also, those mutants that are defective in actin organization (such as *cof1-5* ^*ts*^ and *cof1-8* ^*ts*^) supported TBSV replication at ~4-to-8-fold increased levels at the semi-permissive temperature (Fig 1A and S1A Fig). In contrast, Cof1p mutants that have wt-like organization of actin cytoskeleton [such as *cof1-6*, *cof1-10*, *cof1-11*, *cof1-12*, *cof1-13 and cof1-15;* [66]] did not increase TBSV replication (S1C Fig). We also tested if Cof1p mutants can interact with p33 in yeast using a split-ubiquitin assay. All the Cof1p mutants tested, including *cof1-5* ^*ts*^, *cof1-8* ^*ts*^ and *cof1-20*, interacted with p33 (S1D Fig). Altogether, these data suggest that the ability of Cof1p to organize actin filaments in yeast is important for TBSV replication.

### Mutations in *ACT1* increase TBSV replication in yeast and *in vitro*

**Fig 4:**
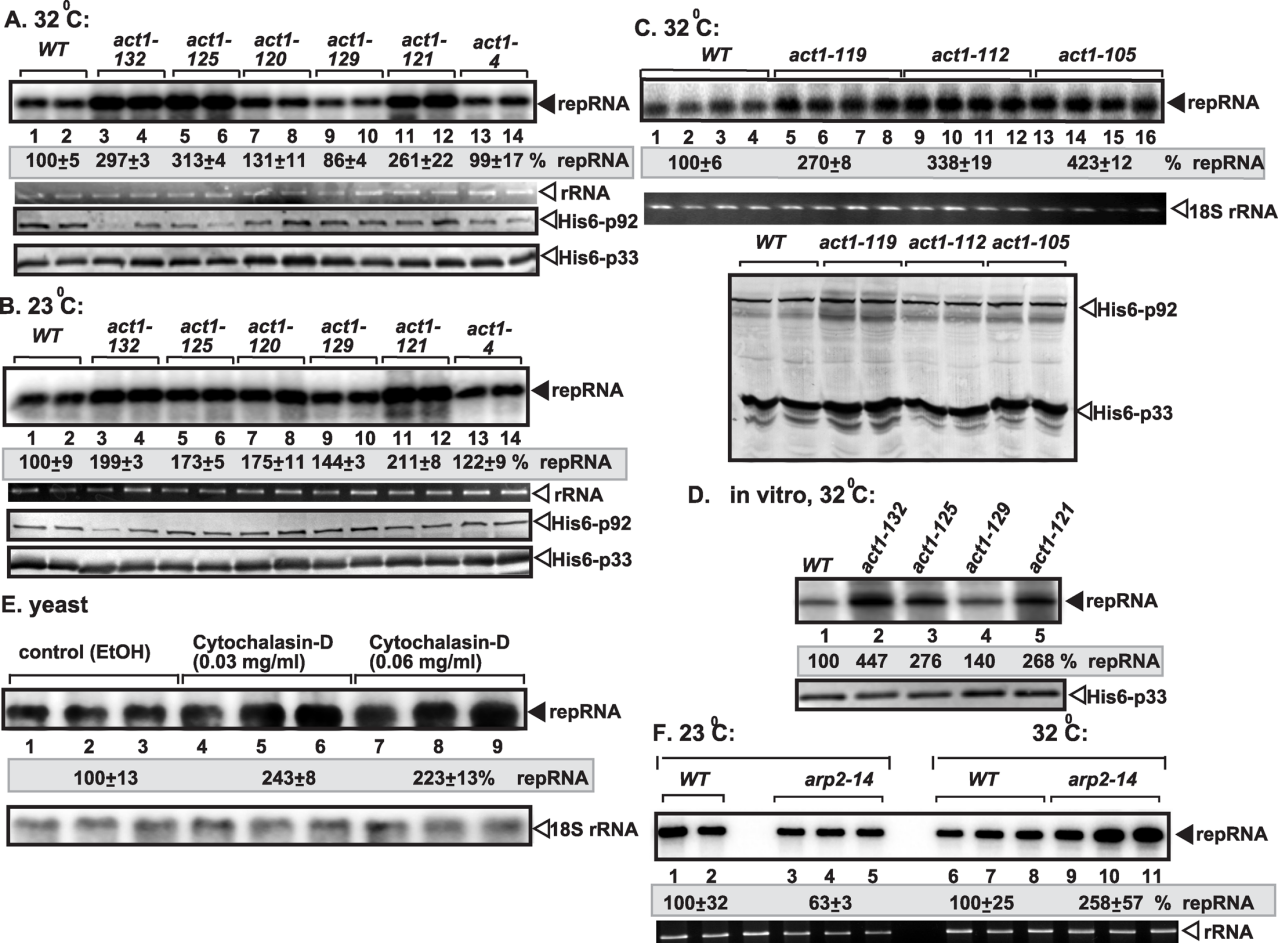
Act1p inhibits TBSV repRNA accumulation in yeast. (A-C) Accumulation of TBSV repRNA in various *act1* ^*ts*^ mutants or wt yeast at the semi-permissive (32°C, panel A), the permissive (23°C, panel B) or the semi-permissive (34°C, panel C) temperatures. To launch TBSV repRNA replication, we expressed His_6_-p33 and His_6_-p92 from the copper-inducible *CUP1* promoter and TBSV DI-72(+) repRNA from the constitutive *ADH1* promoter in the parental (BY4741) and *act1* ^*ts*^ yeast strains. Northern blot analysis was used to detect DI-72(+) repRNA accumulation, which was normalized based on 18S rRNA. Each experiment was repeated three times. Bottom two panels: Western blot analysis of the accumulation level of His_6_-p33 and His_6_-p92 proteins using anti-His antibody. (D) Testing TBSV *in vitro* replication assay based on CFEs. The CFEs were prepared from wt or various *act1* ^*ts*^ yeasts (cultured at the semi-permissive temperatures) expressing the tombusvirus His_6_-p33 and His_6_-p92 replication proteins. Then, the same amount (1 μg) of DI-72(+) repRNA was added to each reaction, followed by *in vitro* tombusvirus replication assay. The denaturing PAGE analysis shows the level of TBSV replication in the various CFEs as indicated. (E) The effect of Act1 inhibitor (Cytochalasin-D) on viral RNA accumulation in wt yeast. Northern blot analysis was used to detect DI-72(+) repRNA accumulation in yeast strain treated with Cytochalasin-D to inhibit Act1p function. Bottom panel: Northern blot analysis of the total ribosomal level in the above yeast samples. (F) Accumulation of TBSV repRNA in *arp2-14* ^*ts*^ mutants or wt yeast at the permissive (23°C) or the semi-permissive (32°C) temperatures. See further details in panel A.

The above data with Cof1p mutants suggested that Cof1p likely inhibits TBSV replication by organizing or remodelling actin filaments due to its ability to promote actin depolymerization and disassembly. To test this model, we used actin mutants, which are defective in actin organization under semi-permissive temperature [67], in the yeast-based TBSV replication assay. Six yeast strains carrying actin ts mutants supported ~3-4-fold higher level of TBSV replication than wt yeast did at the semi-permissive temperature (Fig 4A–4C). These yeast mutants produced p33 and p92 to a comparable level with wt yeast (Fig 4A–4C). Overall, these actin mutants behaved similarly to selected cofilin mutants (Fig 1 and S1 Fig) by supporting high level of TBSV replication, thus suggesting that cofilin and the actin mutants might promote TBSV replication in some similar ways.

Isolation of the membrane fraction of yeasts containing the VRCs, followed by testing for viral RNA synthesis *in vitro*, revealed that the viral replicase from three yeast strains expressing actin ts mutants supported 2-4-fold increased TBSV repRNA replication (Fig 4D). The *in vitro* data are from normalized replication assays that are based on comparable level of p33 replication proteins. Altogether, the *in vitro* data also support that Act1p mutations have similar effects on TBSV replication to Cof1p mutants.

To further demonstrate the active role of the actin network in TBSV replication, we applied Cytochalasin-D inhibitor in yeast, which blocks the polymerization of new actin filaments [59]. We found that the treatment with the inhibitor led to ~2.5-fold increase in TBSV replication (Fig 4E). Similarly, ts mutation in Arp2, which is an actin polymerization factor [58], resulted in ~2.5-fold increase in TBSV replication at the semi-permissive temperature (Fig 4F). These data confirmed that inhibition of new actin filament polymerization needed for dynamic actin remodelling in cells stimulates TBSV replication.

### Inhibition of actin organization increases TBSV replication in plant cells

**Fig 5:**
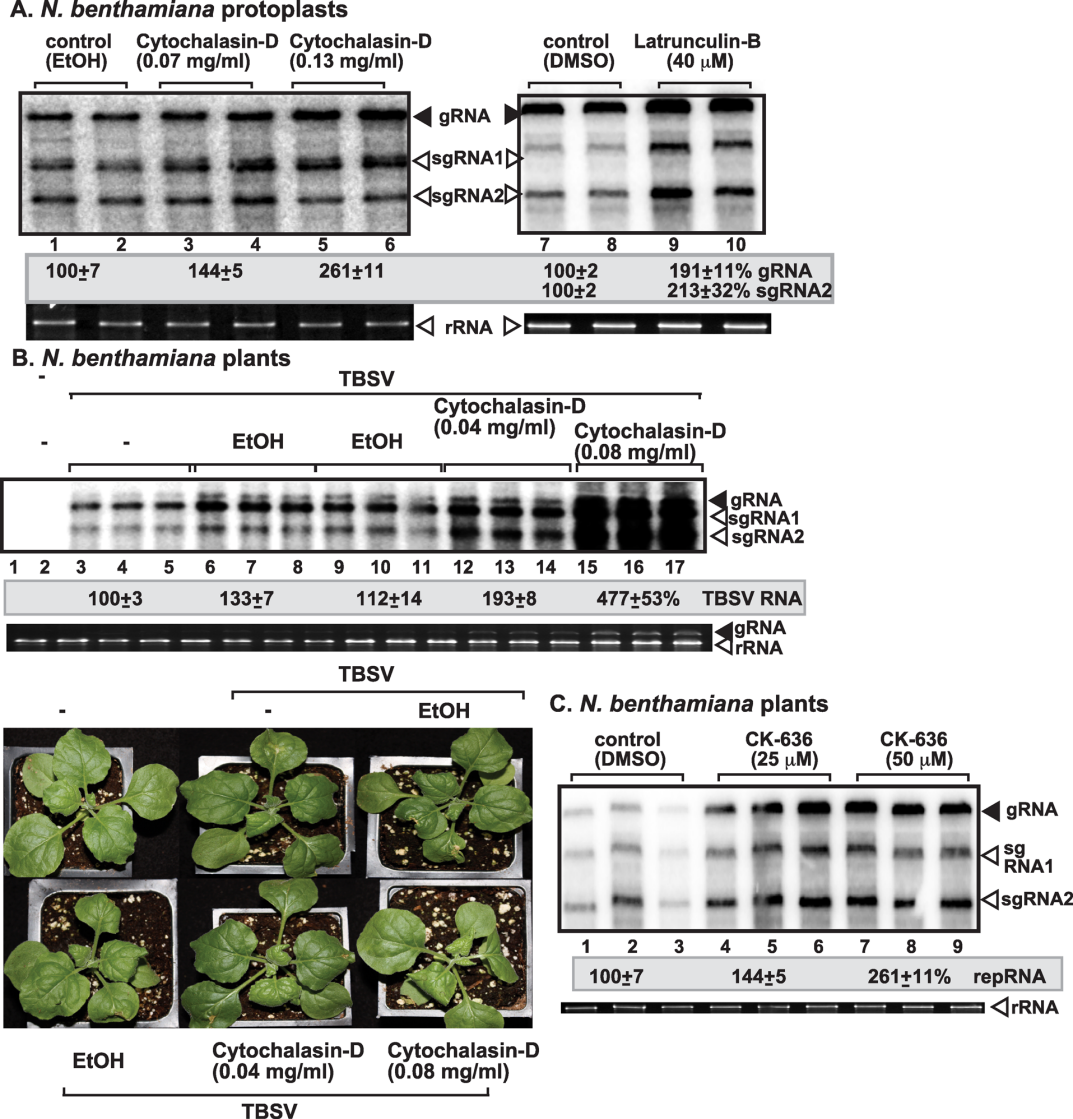
The effect of actin inhibitors on TBSV RNA accumulation in *N*. *benthamiana* protoplasts and plants. (A) Northern blot analysis was used to detect genomic (g)TBSV RNA accumulation in protoplasts treated with Cytochalasin-D (lanes 3–6) and Latrunculin-B (lanes 9–10) to inhibit dynamic actin functions. Protoplasts from *N*. *benthamiana* were electroporated with TBSV gRNA and treated with various concentrations of the inhibitors as shown. Comparable concentrations of the solvents were used as controls. Total RNA samples were obtained 24 hours post-electroporation. The ethidium-bromide stained gel at the bottom shows ribosomal RNA as loading control. The two subgenomic RNAs made during the infection process are also detected. The accumulation level of TBSV RNA was normalized based on the rRNA. Each experiment was repeated three times. (B) The effect of actin inhibitor on TBSV RNA accumulation in *N*. *benthamiana* leaves. Northern blot analysis was used to detect gTBSV RNA. Treatment of *N*. *benthamiana* leaves with Cytochalasin-D promotes the accumulation of TBSV RNAs. Total RNA samples from the inoculated leaves were obtained 3 and 4 days post inoculation and used for Northern blotting (top panel) and gel analysis to show ribosomal RNA level. Symptom intensification caused by TBSV infection in plants treated with Cytochalasin-D. Note that leaf curling is more pronounced in TBSV-infected plants 3 days post-inoculation. (C) The effect of Arp2/Arp3 inhibitor on TBSV RNA accumulation in *N*. *benthamiana* leaves. Northern blot analysis shows gRNA and sgRNAs accumulation. See further details in panel B.

To validate whether our findings with yeast cells on the role of Cof1p and actin in TBSV replication are valid in plant cells, first we tested the effect of Cytochalasin-D and Latrunculin-B inhibitors [68] of actin organization and new actin filament formation on TBSV replication. Treatments of *N*. *benthamiana* protoplasts with these inhibitors led to ~2-to-2.5-fold increase in TBSV genomic (g)RNA accumulation (Fig 5A). These data suggest that dynamic actin network is inhibitory to TBSV replication in single plant cells.

**Fig 6:**
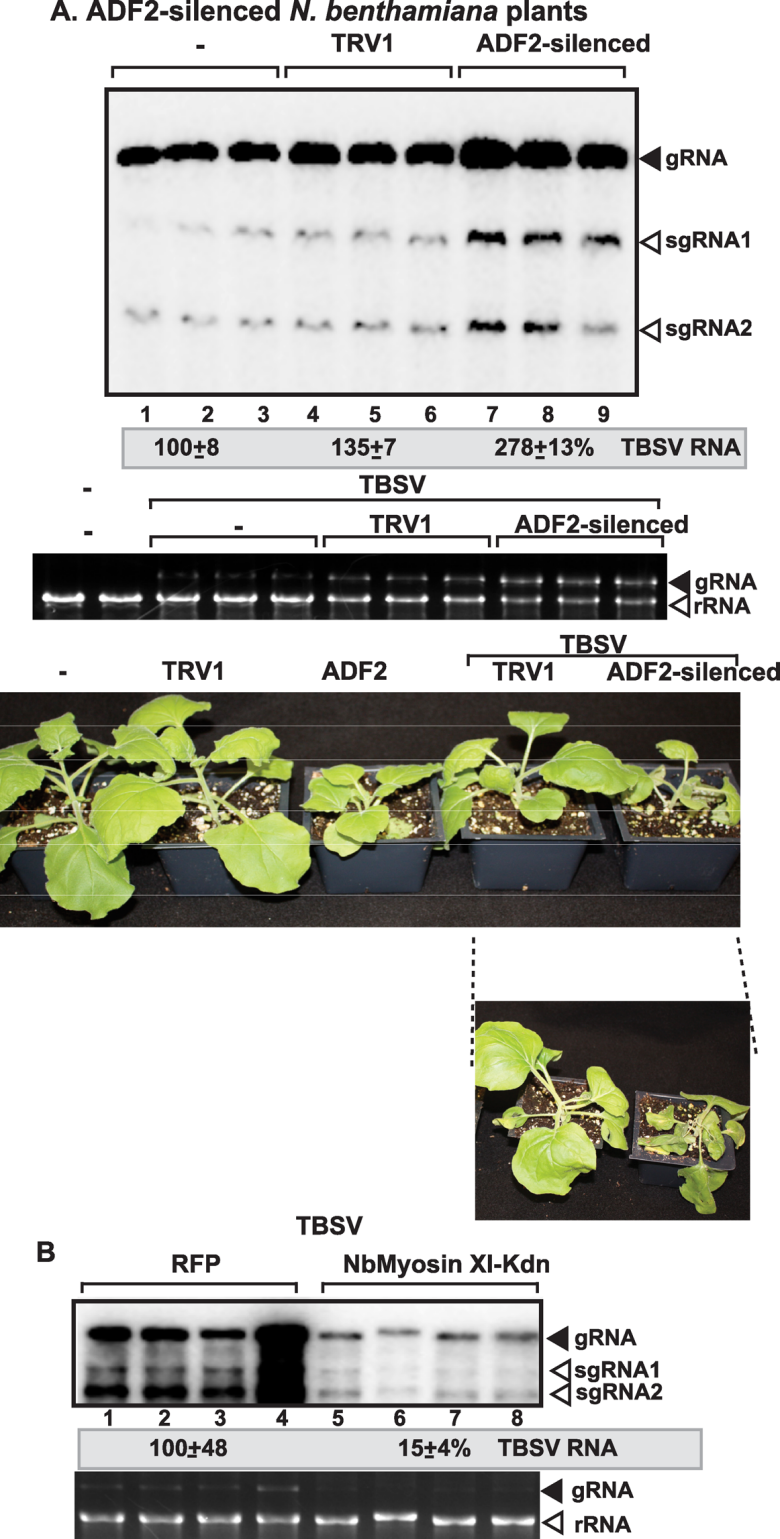
Knock-down of *ADF2* mRNA level by VIGS increases the accumulation of TBSV RNA in *N*. *benthamiana*. (A) Total RNA samples obtained from *N*. *benthamiana* leaves silenced as shown were analyzed by Northern blotting, which shows the accumulation of TBSV gRNA and sgRNAs. Middle image: the ethidium-bromide stained gel shows ribosomal RNA level. We chose the 10th day after VIGS to inoculate the upper, systemically-silenced leaves with TBSV virions. Samples for RNA extractions were taken 4 days post inoculation from the inoculated leaves. Bottom image: Symptoms of TBSV infected *N*. *benthamiana* plants silenced for *ADF2* 14 days after agroinfiltration 4 days after inoculation with TBSV virion preparation. The control experiments included the TRV1 vector as well as infiltration with water (“-”). (B) Expression of dominant negative myosin motor protein inhibits tombusvirus accumulation in *N*. *benthamiana* leaves. The C-terminal tail of myosin XI-K was expressed via agroinfiltration, followed by launching tombusvirus infection 24 h latter via agroinfiltration in the same leaves to minimize the role of cell-to-cell movement in the infiltrated leaves. Samples for Northern blotting were taken 3 days after agroinfiltration with the CNV construct. Expression of RFP via agroinfiltration in *N*. *benthamiana* leaves was used as a contol.

Treatment of whole leaves of *N*. *benthamiana* with Cytochalasin-D also resulted in increased TBSV RNA accumulation by up to ~5-fold in the treated leaves. This was followed by the appearance of symptom intensification in the treated plant compared to the control (ethanol-treated) plants (Fig 5B). Treatment of whole leaves of *N*. *benthamiana* with CK-636, which is an inhibitor of Arp2/3 [69] also resulted in ~2.5-fold increase in TBSV genomic (g)RNA accumulation (Fig 5C). Similarly, knocking down NbAdf2 (the homolog of the yeast Cof1p) by VIGS in *N*. *benthamiana* also led to ~3-fold increase in TBSV gRNA accumulation in infected leaves (Fig 6A). This ultimately led to the rapid death of TBSV-infected *N*. *benthamiana* plants when compared with the empty vector agroinfiltrated plants (Fig 6A). *Arabidopsis* genome codes for four highly conserved *ADF* genes, which are 83-to-88% identical in nucleotide sequences [62–64], making it highly likely that our VIGS approach knocked down the expression levels of all *ADF* genes.

**Fig 7:**
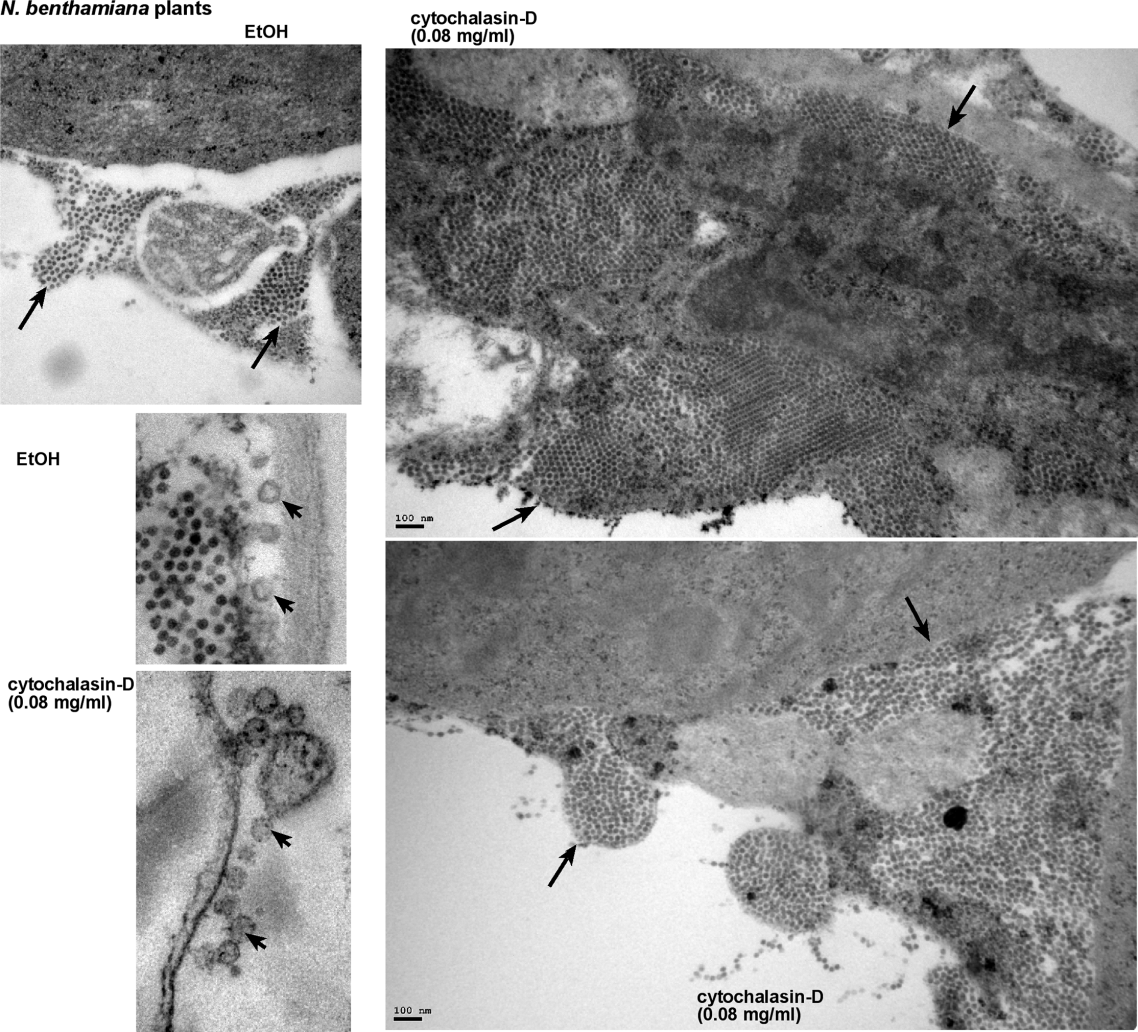
The presence of large number of TBSV virions in *N*. *benthamiana* cells treated with Cytochalasin-D. Representative electron microscopic images of stained ultra-thin sections of *N*. *benthamiana* cells. *N*. *benthamiana* leaves were inoculated with TBSV, and one day later, the same leaves were infiltrated with ethanol or Cytochalasin-D at 80 μg/ml concentration using a syringe to inhibit actin polymerization. Sample preparations from the treated leaves were done two days afterwards. Several characteristic TBSV virion-containing areas are marked with arrows. Close up view of spherules, marked by arrowheads, in plant cells infected with TBSV. EtOH-treated plants were used as a control. Bars in panels represent 100 nm, while the size of the TBSV virions is 30 nm.

Electron microscopy (EM) images of plant cells from whole plants treated with Cytochalasin-D showed large areas of tightly packed virions in the cytosol (Fig 7). Comparable areas in the control plant cells contained fewer numbers of virions, which were packed less tightly together. However, it seems that the sizes of the virus-induced spherules (containing VRCs) were comparable in the Cytochalasin-D- treated and control plant cells (albeit based on only a small number of spherules, Fig 7). The increased number of virions in Cytochalasin-D-treated plant cells was in agreement with the higher accumulation level of TBSV RNAs in Cytochalasin-D-treated than in control plants (Fig 5). Based on all these data, the roles of cofilin (i.e. Adf2 in plants) and actin organization in replication of the TBSV genomic RNA have been firmly established in plants.

### Tombusvirus replication sites are located in the vicinity of actin filaments in yeast and plant cells

**Fig 8:**
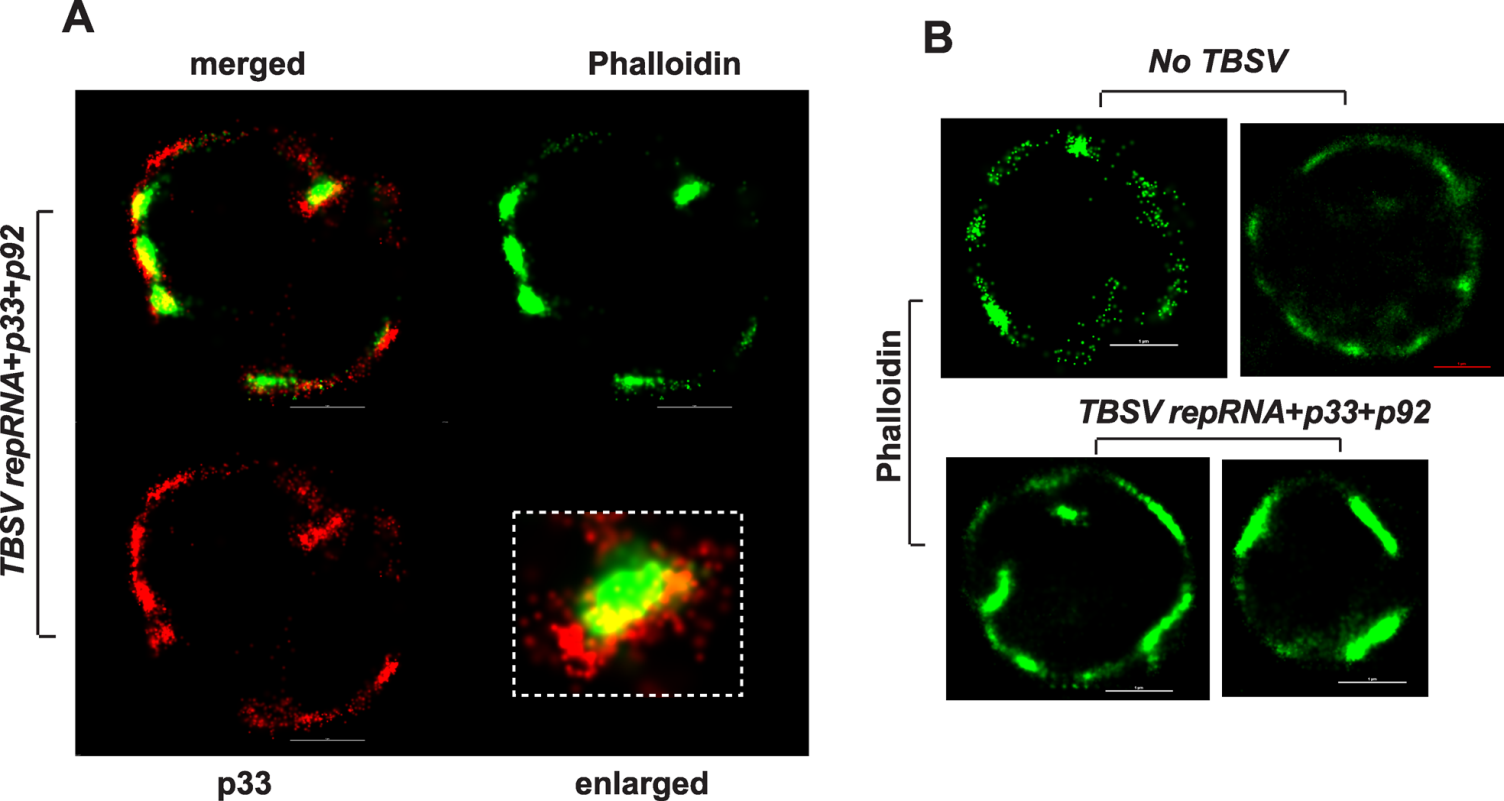
Super Resolution laser microscopic images of yeast cells. (A) A yeast cell replicating TBSV repRNA was imaged based on Alexa Flour 647 (for p33 replication protein) and for ATTO488-phalloidin, which detect actin filaments. The bars represent 1 μm. The boxed area represents enlargement of the image to visualize the localization of actin and p33 replication protein in yeast. (B) Yeast cells free of viral components (top images) or replicating TBSV repRNA (bottom images) were imaged based on ATTO488-phalloidin, which detect actin filaments. The bars represent 1 μm. The images in both panels were prepared by a Nikon Super Resolution Microscope N-STORM and image processing was performed using NIS-element software.

To study the putative connection between the actin network and tombusvirus replication sites, we performed super-resolution microscopy on wt yeast cells actively replicating TBSV repRNA. The replication sites were detected with anti-p33 antibody, which also recognizes p92^pol^ because of the overlapping sequence in the pre-readthrough region in p92. The actin filaments were detected by phalloidin (F-actin specific reagent) attached to Atto-488 dye. Interestingly, several large tombusvirus replication sites were located in the vicinity of the actin filaments in wt yeast (Fig 8A and S1 Video). Also, the actin filaments formed larger structures (called “patches” with up to 2-fold increase in diameter) in yeast cells replicating TBSV than in the absence of viral components (Fig 8B).

**Fig 9:**
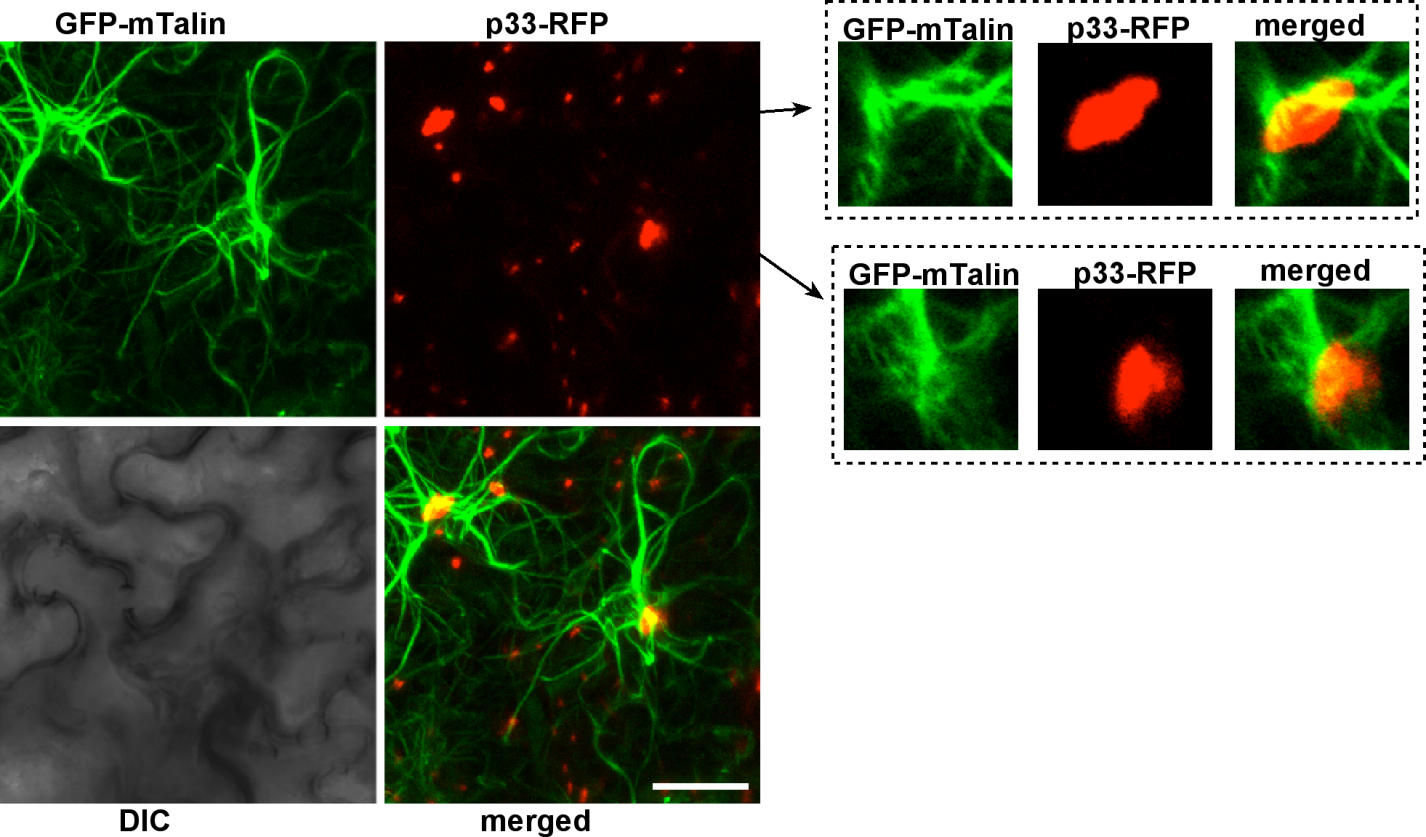
Confocal laser microscopic images of plant cells expressing TBSV p33 replication protein. The GFP-mTalin transgenic *N*. *benthamiana* plants were agro-infiltrated with a plasmid expressing p33-RFP. The images on the left represent Z-stack images (overlay of individual images), while on the right, represent single plane images. Note the localization of large p33-RFP containing areas (i.e., replication organelles) at the intersection of actin cables. The bar represents 20 μm.

**Fig 10:**
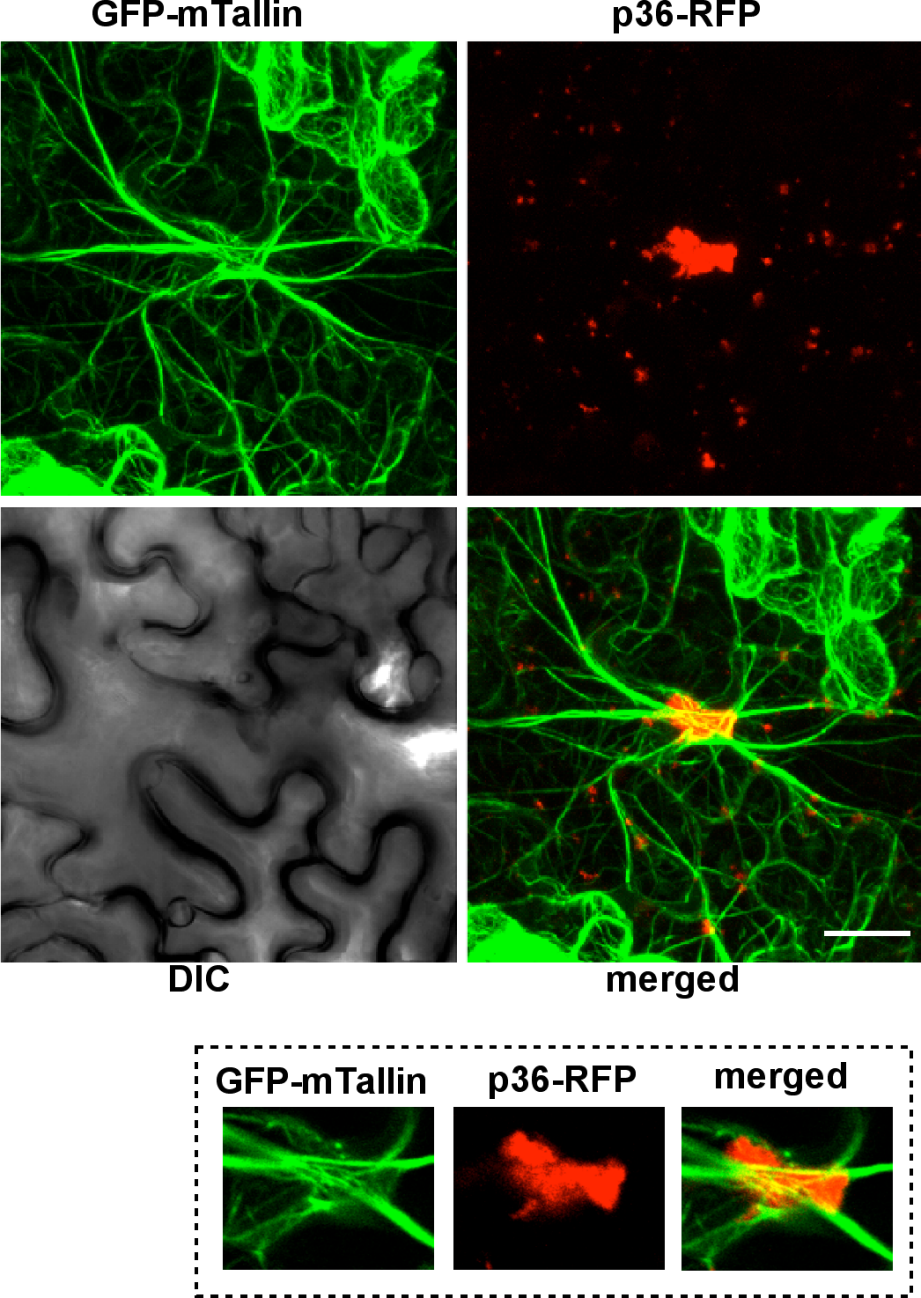
Confocal laser microscopic images of plant cells expressing CIRV p36 replication protein. The GFP-mTalin transgenic *N*. *benthamiana* plants were agro-infiltrated with a plasmid expressing the CIRV p36-RFP. The images on the top represent Z-stack images, while on the bottom, represent single plane images. Note the localization of large p36-RFP containing areas (i.e., replication organelles including mitochondria) at the intersection of actin cables. The bar represents 20 μm.

Because the actin cables are difficult to visualize in the tiny yeast cells, we also used plant cells transiently expressing RFP-tagged p33 replication protein. The actin cables were visualized via transgenically expressed GFP-tagged mTalin, which binds to actin filaments [70]. Confocal laser microscopy imaging revealed that the largest TBSV replication sites (marked by p33-RFP), which are known to form on aggregated peroxisomal membrane surfaces, were formed where actin cables intersected (Fig 9). Individual cross-sections showed that the actin cables surrounded and sometimes crossed through the large viral replication sites/organelles (Fig 9, images on the right, and S3 Fig, which shows the structures in the presence of TBSV infection). Interestingly, we observed very similar arrangements between actin cables and viral replication sites (marked by p36-RFP) in case of *Carnation Italian ringspot virus* (CIRV), a closely related tombusvirus that replicates on mitochondrial membrane surfaces (Fig 10 and S4 Fig, which shows the structures in the presence of CIRV infection). Comparison of actin filament network revealed overall higher density and thicker and larger number of individual filaments in TBSV-replicating (S3 Fig versus S5 Fig), and to a lesser extent, CIRV-replicating plant cells (S4 Fig) than in uninfected control cells (S5 Fig). Thus, the actin network plays a role in formation of tombusvirus replication sites that resemble replication organelles in plant cells [71].

The close association between the actin network and tombusvirus replication proteins in both yeast and plant cells indicated a putative functional role for actin in tombusvirus replication. Since both cofilin and actin are essential for yeast and plant cells, in the above experiments we used approaches that temporarily and partially blocked the dynamic actin network, but still allowed the function of the stable actin network. To test the putative role of the actin network, we inhibited the functions of both dynamic and stable actin networks via expressing dominant negative mutant of myosin XI-K that is a motor protein involved in actin-based moving of cargos in the cytosol of plant cells [72,73]. Interestingly, expression of a dominant negative mutant of myosin XI-K strongly inhibited tombusvirus replication in *N*. *benthamiana* leaves (Fig 6B). All these data support the functional role of the actin network in tombusvirus replication.

### Tombusvirus replication takes advantage of altered sterol transport in yeast with mutant cofilin or actin

**Fig 11:**
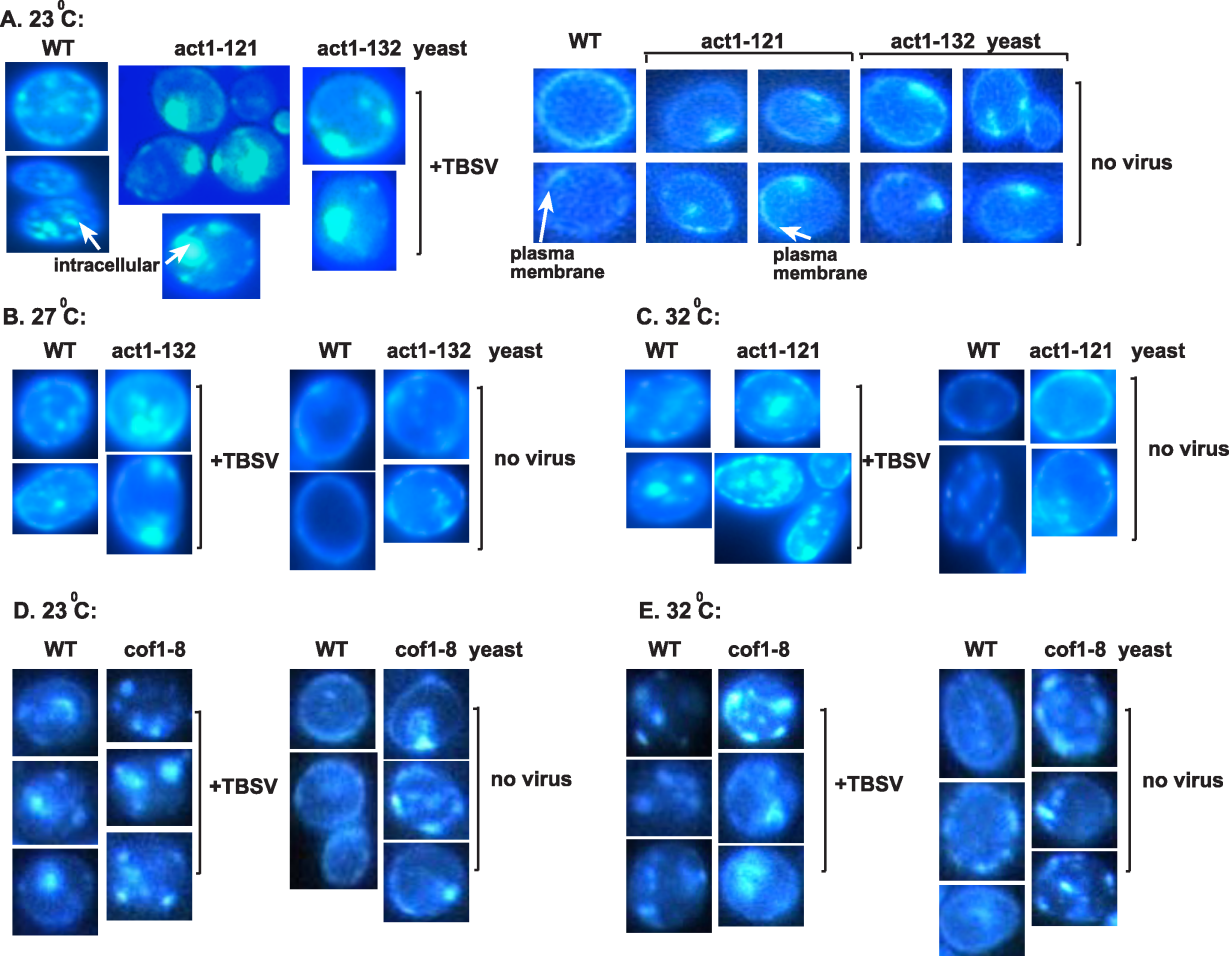
The cofilin and actin mutants facilitate the enrichment of sterols at the sites of tombusvirus replication in yeast. (A) Re-localization of ergosterols to internal punctate structures in yeast replicating TBSV repRNA. Fluorescent microscopic images of yeast cells stained with filipin dye. Note that filipin stains ergosterols present mostly at the plasma membrane in virus-free wt yeast cells, while *act1-121* ^*ts*^ yeast and *act1-132* ^*ts*^ yeast show uneven distribution of ergosterols in the absence of viral components (right images). (B) Similar experiments as in Panel A, except performed at the semi-permissive temperature (27°C) in *act1-132* ^*ts*^ and WT yeasts. (C) Similar experiments as in Panel A, except performed at the semi-permissive temperature (32°C) in *act1-121* ^*ts*^ and WT yeasts. (D) Re-localization of ergosterols to internal punctate structures in yeast replicating TBSV repRNA in *cof1-8* ^*ts*^ or wt yeasts at the permissive temperature. Fluorescent microscopic images of yeast cells stained with filipin dye. (E) Re-localization of ergosterols to internal punctate structures in yeast replicating TBSV repRNA in *cof1-8* ^*ts*^ or wt yeasts at the semi-permissive temperature. See further details in panel A. Each experiment was repeated at least three times.

Because TBSV replication greatly depends on the virus-driven retargeting of sterols, which become highly enriched at viral replication sites and facilitate the efficient assembly of VRCs [45,47], we analyzed the sterol distribution in cofilin and actin mutant yeasts. Filipin-based staining of sterols, which can be visualized by fluorescent microscopy [45], revealed the formation of large sterol-enriched compartments in cofilin and actin mutant yeasts when compared to wt yeast showing smaller sterol-enriched compartments (Fig 11). The difference in sterol enrichment and distribution was especially striking in the presence of viral components in *cof1-8* ^*ts*^ (Fig 11E), *act1-132* ^*ts*^ and *act1-121* ^*ts*^ (Fig 11B and 11C) yeasts in comparison with the wt yeast at the semi-permissive temperature. We have shown previously that these internal (not plasma membrane localized as observed in the absence of viral components in wt yeast, Fig 11A) sterol-enriched compartments represent the viral replication sites [45].

In comparison with the wt yeast, the cofilin and actin mutant yeasts showed less even distribution of sterols in the plasma membrane, suggesting defect in normal sterol transport in case of the cofilin and actin mutants (Fig 11). Based on these observations, we suggest that TBSV could take advantage of the defective sterol transport mechanism in cofilin and actin mutant yeasts that likely allows the virus to easily hijack sterols and enrich them at the sites of viral replication that leads to highly efficient VRC assembly and increased viral RNA synthesis.

**Fig 12:**
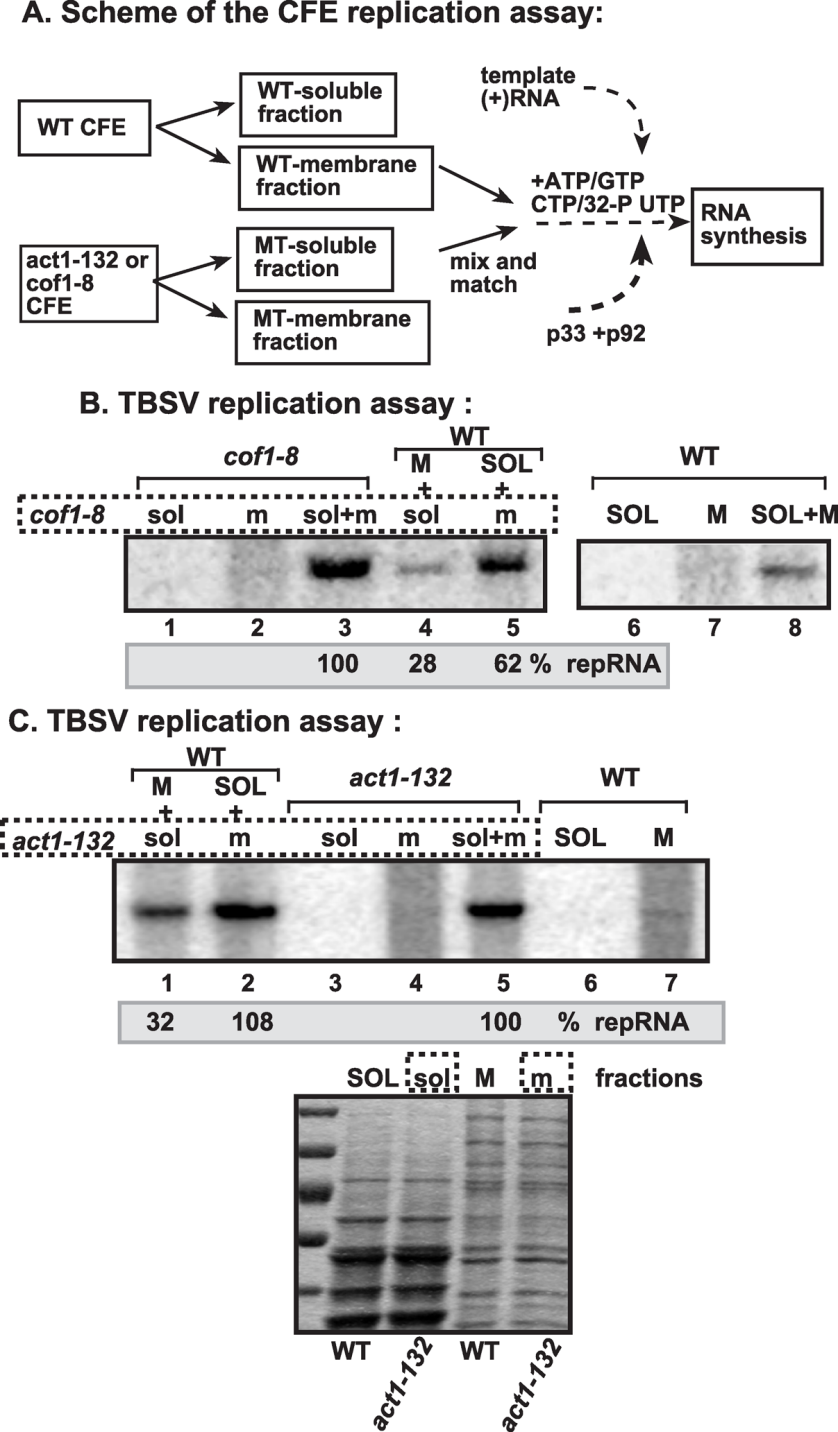
The membrane fraction of cofilin and actin mutant yeasts is critical for tombusvirus replication *in vitro*. (A) The scheme of the CFE-based TBSV replication assay. The membrane and soluble fractions of the CFEs prepared from wt, *cof1-8* ^*ts*^ or *act1-132* ^*ts*^ yeasts were used alone or in combinations (as shown). Purified recombinant MBP-p33 (7 pmol) and MBP-p92^pol^ (4 pmol) replication proteins in combination with DI-72 (+)repRNA (0.5 μg) were added to the CFEs. (B) Denaturing PAGE analysis of the ^32^P-labeled TBSV repRNA products obtained is shown. Note that, prior to the TBSV replication assay, the membrane and soluble fractions of CFEs derived from wt or *cof1-8* ^*ts*^ yeasts were adjusted to contain comparable amounts of the cellular proteins. Note that the enhanced TBSV repRNA replication in CFE prepared from *cof1-8* ^*ts*^ yeast depends on the membrane fraction. (C) Comparable CFE-based TBSV replication studies as in panel B, except using CFE prepared from *act1-132* ^*ts*^ yeast. Bottom image shows coomassie-stainde SDS PAGE of the total proteins in the membrane and soluble fractions of CFEs. Note that the enhanced TBSV repRNA replication in CFE prepared from *act1-132* ^*ts*^ yeast depends on the membrane fraction.

To examine if the membrane fraction from the *cof1-8* ^*ts*^ or *act1-132* ^*ts*^ yeasts is more suitable for TBSV replication than from wt yeast at the semi-permissive temperature, we used a mix-and-match CFE approach as shown schematically in Fig 12A. The combination of membrane fraction from *cof1-8* ^*ts*^ with the soluble fraction from the wt yeast (Fig 12B, lane 5) supported TBSV repRNA replication almost as efficiently as the nonfractionated CFE from *cof1-8* ^*ts*^ (Fig 12B, lane 3). On the contrary, the combination of membrane fraction from wt yeast with the soluble fraction from *cof1-8* ^*ts*^ supported TBSV repRNA replication at a ~4-fold reduced level (Fig 12B, lane 4), suggesting that the membrane fraction of *cof1-8* ^*ts*^ is responsible for the enhanced TBSV replication *in vitro*. We obtained similar results with the membrane fraction of *act1-132* ^*ts*^ (Fig 12C, lane 5), which was responsible for the enhanced level of TBSV repRNA replication *in vitro* (Fig 12C). Altogether, these *in vitro* data are in agreement with the model that the intracellular membranes in *cof1-8* ^*ts*^ or *act1-132* ^*ts*^ yeasts are more suitable for TBSV replication, possibly because they are sterol-rich, in comparison with the intracellular membranes in wt yeast.

### Efficient recruitment of oxysterol binding protein and VAP protein by TBSV in yeast with mutant cofilin or actin

TBSV controls intracellular sterol transport via hijacking oxysterol binding proteins (OSBP-like or ORP, such as Osh3p, Osh5p, Osh6p and Osh7p) and VAP protein (VAMP-associated protein, such as yeast Scs2p or Vap27-1, a plant ortholog) via interaction with the p33 replication protein [45]. These cellular proteins help TBSV to stabilize membrane contact sites (MCSs) between the ER and the peroxisome, thus facilitating the efficient transport of sterols from the ER to the peroxisomal membranes where a large number of VRCs forms [45].

**Fig 13:**
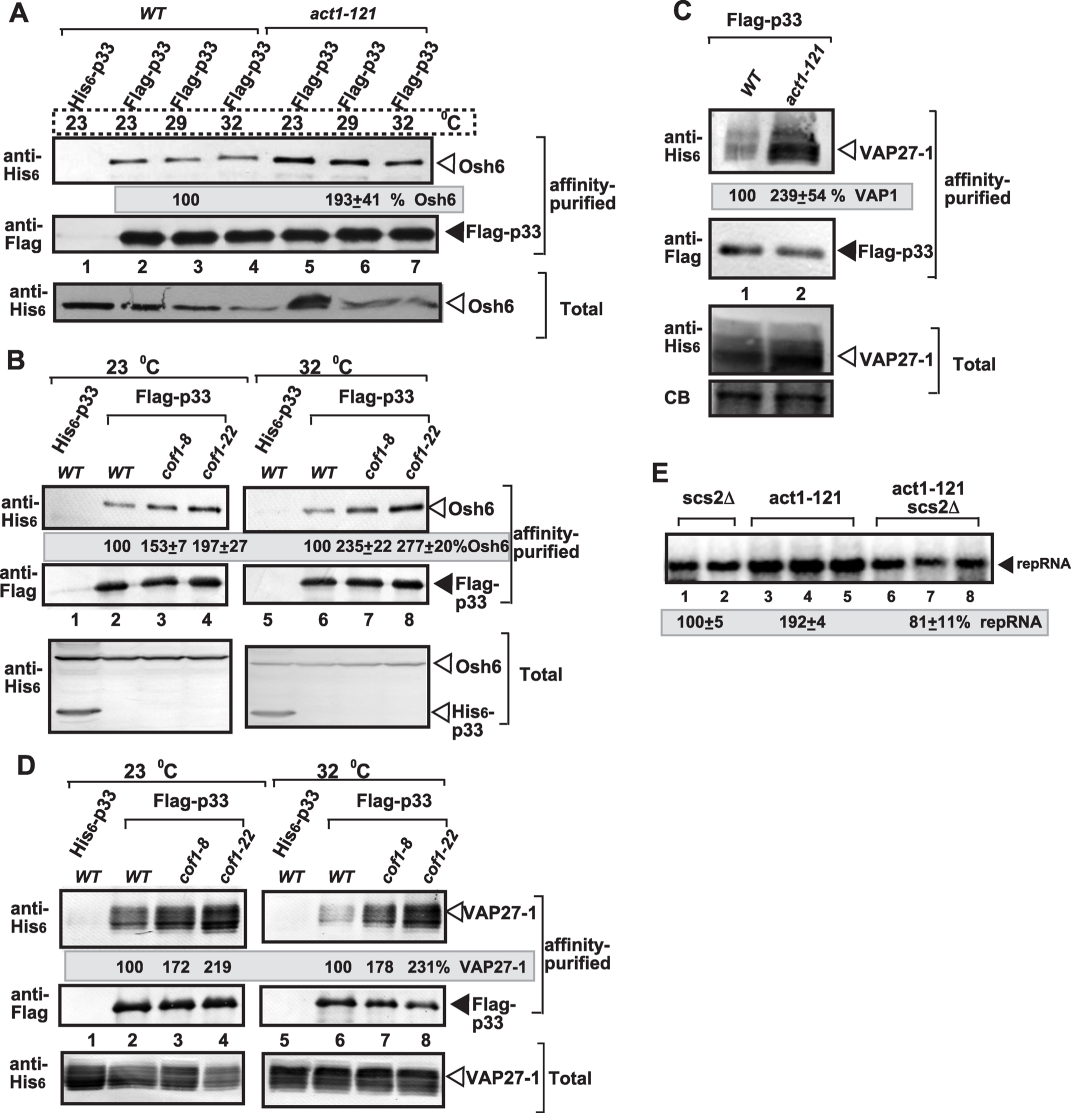
Cof1p and Act1p mutations affect the subversion of MCS forming OSBP-like Osh and VAP proteins for tombusvirus replication in yeast. (A) Enhanced co-purification of yeast Osh6p ORP with the p33 replication protein from *act1-121* ^*ts*^ yeast in comparison with WT yeast. The FLAG-tagged p33 and FLAG-p92 were co-purified from solubilized membranous fraction of yeast extracts using a FLAG-affinity column. Yeast expressing the His_6_-tagged p33 was used as a control. Top panel: Western blot analysis of the co-purified 6xHis-tagged Osh6p with anti-His antibody in the affinity-purified preparations. Middle panel: Western-blot analysis of the same samples as in the top panel, but using anti-FLAG antibody. Bottom panels: Western blot analysis of 6xHis-Osh6p with anti-His antibody in the total protein extract from yeast expressing the shown proteins. (B) Enhanced co-purification of yeast Osh6p ORP with the p33 replication protein from *cof1-8* ^*ts*^ yeast in comparison with WT yeast at the permissive and semi-permissive temperatures. See further details in panel A. (C) Enhanced co-purification of the ER-localized Vap27-1 with the p33 replication protein from *act1-121* ^*ts*^ yeast in comparison with WT yeast at the semi-permissive temperature. The FLAG-affinity purification was done as in panel A. Top panel: Western blot analysis of the co-purified 6xHis-Vap27-1 with anti-His antibody in the affinity-purified preparations. Middle panel: Western-blot analysis of the same samples as in the top panel, but using anti-FLAG antibody. Bottom panels: Western blot analysis of 6xHis-Vap27-1 with anti-His antibody in the total protein extract from yeast expressing the shown proteins. CB: Coomassie-stained SDS-PAGE of total protein extract. Each experiment was repeated two times. (D) Enhanced co-purification of the ER-localized Vap27-1 with the p33 replication protein from *cof1-8* ^*ts*^ yeast in comparison with WT yeast at the permissive and semi-permissive temperatures. See further details in panel A. (E) Dependence of TBSV repRNA replication on Scs2p VAP protein in yeast. The double-mutant yeast carrying *act1-121* ^*ts*^ mutation and missing *SCS2* shows reduced replication of TBSV repRNA based on Northern blot analysis. See further details in Fig 1.

To test if TBSV could efficiently co-opt Osh6p and Vap27-1 [45] in yeast cofilin or actin mutants, we purified p33 replication proteins from detergent-solubilized membrane fractions, followed by Western blotting to measure the co-purified cellular Osh6p and Vap27-1. We found ~2-to-2.5-fold increase in the co-purified cellular Osh6p in *cof1-8* ^*ts*^, *cof1-22* ^*ts*^ (Fig 13B), *act1-121* ^*ts*^ (Fig 13A) and *act1-132* ^*ts*^ (Table 1) in comparison with wt yeast at the semi-permissive temperature. The Osh6p protein was also more efficiently co-purified with p33 in *act1-105* ^*ts*^, and *act1-112* ^*ts*^ yeasts in comparison with wt yeast at the permissive or semi-permissive temperature (S6 Fig). Interestingly, we also found ~2-to-2.5-fold increase in the co-purified cellular Vap27-1, in *cof1-8* ^*ts*^, *cof1-22* ^*ts*^ (Fig 13D), *act1-121* ^*ts*^ (Fig 13C) and *act1-132* ^*ts*^ (Table 1) in comparison with wt yeast at the semi-permissive temperature. Altogether, the more efficient co-purification of Osh6p and Vap27-1 in the cofilin and actin mutants suggests the enhanced or more stable formation of viral-induced MCSs and could explain the high enrichment of sterols at internal sites (likely representing the replication sites) in the cofilin and actin mutants.

**Table 1:** Co-purified host proteins with p33 from act1-132 yeast.

*Protein*	*percentage*
***Ded1 helicase***	***108±9***
***Cdc34 E2 enzyme***	***207±22***
***Osh6 ORP***	***258±37***
***Pbp2 RNA binding***	***85±18***
***Rpn11 deubiquitinase***	***161±12***
***Tdh2 GAPDH***	***111±15***
***Tef1 eEF1A***	***96±8***
***Vap27-1 (Scs2-like)***	***218±33***
***Vps4 AAA ATPase***	***53±6***
***Vps23 ESCRT I***	***108±9***

Co-purification of the given host protein with p33 from WT yeast is taken as 100%.

To further test if indeed the actin mutants affect sterol-enrichment at viral replication sites, we deleted Scs2p VAP protein in yeast (double mutant *act1-121* ^*ts*^ *scs2*Δ yeast), followed by measuring TBSV repRNA replication. Interestingly, the double mutant supported a reduced level of repRNA replication, comparable to that observed in the single mutant yeast (*scs2*Δ) (Fig 13E). These data suggest that the actin mutant could enhance TBSV replication only when functional VAP protein is present, thus when MCSs are formed, in *act1-121* ^*ts*^ yeast.

The co-purification-based proteomics approach also revealed that co-opted host factors, such as Cdc34 ubiquitin-conjugating enzyme, Rpn11 deubiqutinase and Vps23p ESCRT factor, were co-purified with the tombusvirus replicase by ~2-fold more efficiently from *act1-132* ^*ts*^ yeast in comparison with wt yeast (Table 1). Other cellular host factors, such as Tef1p (eEF1A), Tdh2p (GAPDH), DDX3-like Ded1p and Vps4p AAA ATPase, were co-purified with p33 as efficiently from *act1-132* ^*ts*^ yeast as from wt yeast (Table 1). Based on these data, we suggest that the actin mutations also facilitate the subversion of some cellular host factors by TBSV into the VRCs.

## Discussion

Tombusvirus replication involves the assembly of hundreds-to-thousands of VRCs in infected cells that leads to robust viral RNA synthesis [45,71]. The VRCs are clustered in large viral-induced compartments, called replication organelles [74]. The formation of individual VRCs, housed in spherule-like membranous structures, requires p33 and p92 replication proteins, the viral (+)RNA and 15–20 co-opted host proteins, lipids, such as sterols and phospholipids, and likely metabolites [3,16,43]. TBSV replication also depends on MCSs to transfer sterols to the sites of replication, which is the peroxisomal membrane for TBSV and mitochondrial outer membrane for CIRV [45]. How can all these viral and cellular components come together at the sites of replication and facilitate the formation of large replication organelles containing relatively stable membranous VRCs inside the cytoplasm? Based on the current work, it seems that the actin network plays a key role in this critical viral process. Accordingly, the large p33 containing replication structures are located in the close vicinity of actin patches in yeast cells or around actin cable hubs in infected plant cells (Figs 8–10). Therefore, these actin filaments are likely involved in VRC assembly and the formation of large viral replication organelles containing many individual VRCs. Indeed, expression of dominant negative mutant of myosin XI-K motor protein, which is involved in actin-based intracellular cargo movement [72,73], strongly inhibited tombusvirus accumulation in *N*. *benthamiana* leaves, supporting the functional role the actin network in tombusvirus replication (Fig 6B).

Yet, several mutations in the essential *ACT1* gene or the use of actin inhibitors enhance TBSV replication in yeast, suggesting that *ACT1* is an antiviral restriction factor. However, the identified mutations and the inhibitors affect the assembly of new actin filaments without blocking the functions of the existing actin filaments [67,75]. Therefore, we propose that TBSV do utilize the existing actin filaments to concentrate host and viral components at replication sites and assemble VRCs. Accordingly, the large tombusvirus replication organelles are frequently formed where the actin cables meet in the cytosol for both peroxisomal TBSV and the mitochondrial CIRV in plant cells (Figs 9 and 10 and S3–S5 Figs). On the other hand, the dynamic rearrangement of the actin filaments might limit the VRC assembly process or used in antiviral responses by the cell, making new actin filament formation inhibitory to TBSV replication. Altogether, the emerging picture from all our data is that the dynamic actin network functions as a virus restriction factor by inhibiting tombusvirus replication, while the stable actin network provides pro-viral function in tombusvirus VRC formation. This could be a more general phenomenon, because disruption of the actin network leads to enhanced susceptibility to pathogenic bacteria due to inhibition of the innate immunity response of the plant [76].

### Interaction between the cellular cofilin and viral replication factor regulates actin organization and promotes TBSV replication

Using tombusviruses, we have discovered that the cellular cofilin actin depolymization factor is targeted by TBSV to obstruct the dynamic actin network. Down-regulation of Cof1p level or the use of Cof1p ts mutants at the semi-permissive temperature lead to increased level of TBSV RNA accumulation in yeast cells. The increased RNA replication is due to highly active VRCs, based on testing the *in vitro* activity of replicase in CFE prepared from yeast expressing *cof1-8* ^*ts*^, which was ~3-fold more active than the replicase operating in wt CFE (Fig 1D). Also, expression of NtAdf2, a plant homolog of Cof1p [62,63] reduced TBSV replication *in vitro* (Fig 2D).

**Fig 14:**
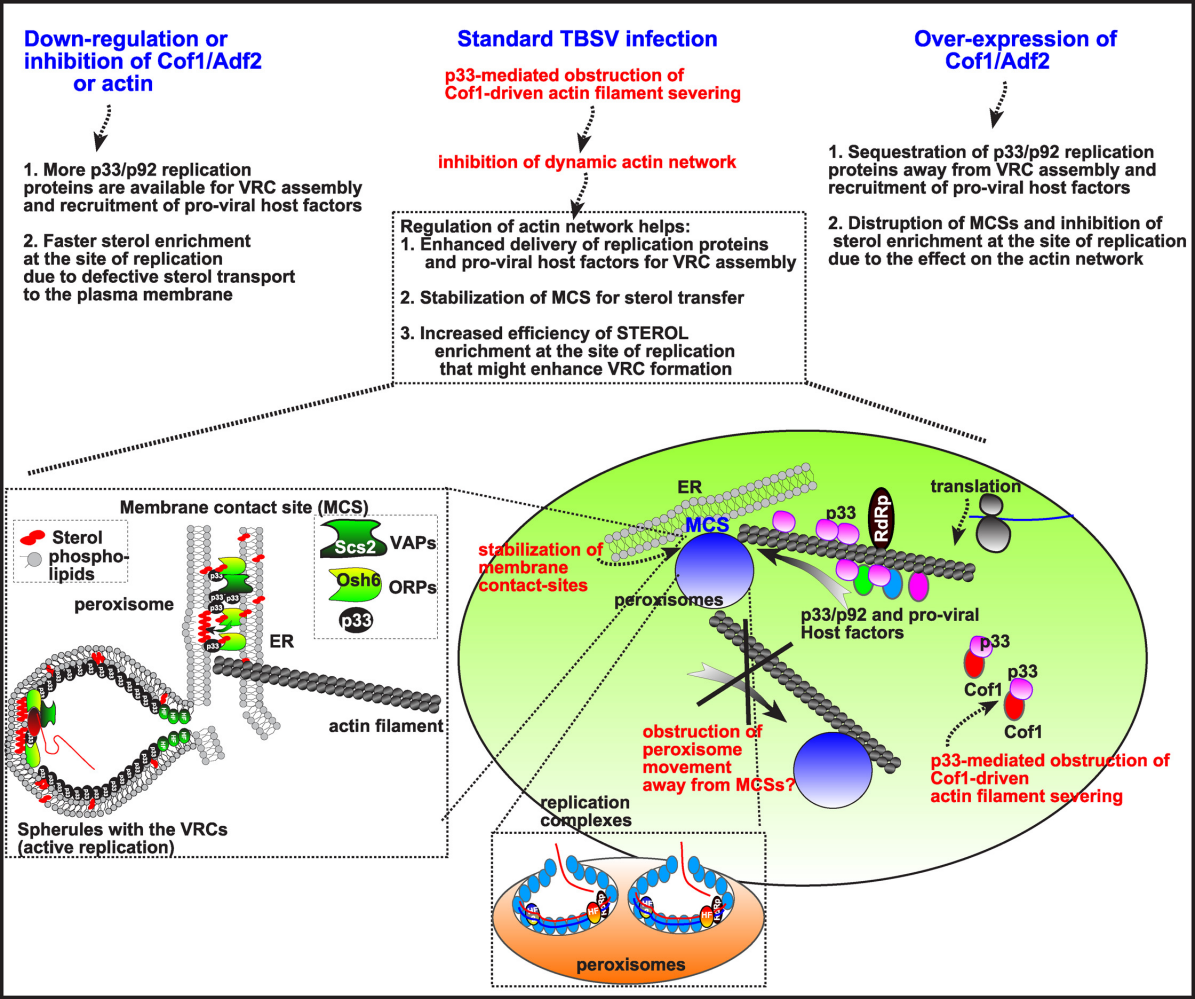
Summary and model on the role of p33 replication protein driven obstruction of the cellular actin network in TBSV replication. Top: three scenarios with different levels of Cof1p are shown, including the predicted effects on TBSV replication. Bottom: The formation of VRCs (virus-induced membrane invaginations, called spherules) in the peroxisomes requires the subversion of cellular co-factors and lipids, whose recruitment could be enhanced by obstructing new actin filament formation via p33:Cof1p interaction. In addition, formation and/or stabilization of membrane contact sites (MCS) between ER and peroxisome might be promoted by blocking the dynamic actin network, and halting the movement of peroxisomes away from ER to various subcellular locations. Moreover, obstructing the dynamic actin network might also facilitate localized enrichment of sterols within VRCs, which is known to be required for tombusvirus VRC formation. Left: Enlargement of the scheme showing the predicted MCS with co-opted cellular ORP and VAP proteins that facilitate tombusvirus replicase assembly (shown as a vesicle-like structure) [45]. The OSBP-like ORP oxysterol-binding proteins are recruited via binding to the tombusviral p33 replication protein to the ER-bound Scs2p VAP to form MCS between the ER and peroxisomal membranes. The ORPs then facilitate the enrichment of sterols in the peroxisomal membrane, forming sterol-rich microdomains needed for the formation of virus-induced spherules. These spherules contain the VRCs performing viral RNA synthesis.

It seems that the inhibitory effect of Cof1p on TBSV replication is due to two different mechanisms. First, the interaction between Cof1p (Adf2 homolog) and p33 and possibly p92 could partially sequester the replication proteins, thus limiting their participation in VRC formation and TBSV RNA replication. Second, Cof1p also seems to inhibit TBSV replication via facilitating the dynamic actin re-organization in live yeast cells. The Cof1p-driven disassembly of existing actin filaments in combination with formation of new actin filaments might inhibit the continuous growth of the replication compartments, which could depend on stable MCSs for sterol transfer/enrichment at the sites of replication and steady supply of host factors to the replication sites (Fig 14). Accordingly, yeasts expressing Act1p ts mutants at the semi-permissive temperature supported increased level of TBSV RNA accumulation (Fig 4). Moreover, pharmacological inhibition of actin organization by Cytochalasin D or Latrunculin B in yeast, plant protoplasts or whole plants also resulted in increased level of TBSV RNA accumulation (Figs 4–7). Thus, actin organization, especially the formation of new actin filaments is a major restriction factor for virus replication and TBSV modulates the dynamic actin network to obstruct the formation of new actin polymers.

How does dynamic actin organization limit TBSV replication in live cells? Based on our current knowledge on TBSV replication, 15–20 cytosolic pro-viral host factors, peroxisomes/ER, phospholipids and sterols are required for efficient TBSV replication [9,16,17,46,47,77–79]. We predict that TBSV could easily co-opt these host factors for efficient VRC assembly when dynamic actin organization and remodeling of the actin network is inhibited. The obstructed actin network might inhibit the normal cellular distribution and function of host proteins and organelles, which might become more readily accessible to TBSV when new actin filament formation is hindered (Fig 14). Indeed, formation of large replication foci in combination with increased replicase activity is seen in *cof1-8* ^*ts*^ yeast at the semi-permissive temperature (Fig 3). Similar observation of the presence of large replication foci and increased replication was documented for yeast over-expressing Ino2p, a transcription factor regulating phospholipid biosynthesis [46]. Therefore, our model predicts that it is more difficult for the virus to recruit the host factors, sterols and peroxisomes for efficient VRC assembly when dynamic actin organization facilitates rapid transport of host components to their final destination within the cell, while disruption of actin organization via p33-Cof1p interaction and Cof1p re-localization (in natural infections) or via inhibitors, actin or Cof1p mutations enhances VRC assembly and TBSV replication (Fig 14). The actin network might also regulate the availability of given host factors, such as eEF1A, whose function is affected by actin through promoting GTP hydrolysis by the GTP-bound eEF1A and subsequent release of eEF1A from translation [80] that might increase the availability of eEF1A for viral functions [38,81].

Cofilin not only regulates actin dynamics, but also affects phospholipid metabolism via regulating phospholipase D activity [53,54,56,57]. However, previous genomics and proteomics screens with TBSV have not identified *SPO14* or other phospholipase genes [9,26–30,32,34]. Therefore, the evidence is lacking for this mechanism during TBSV replication. Also, p33 interaction with Cof1p might interfere with apoptosis that requires re-localization of cofilin to the mitochondria [54].

Since other (+)RNA viruses also remodel subcellular membranes and rewire cellular pathways to facilitate virus replication and avoid antiviral responses [74], it is possible that targeting cofilin and actin could be wide-spread among viruses. Accordingly, during HIV infection, the viral Nef protein inactivates cofilin molecules via its interaction with Pak2 cellular kinase that phosphorylates cofilin [82]. This results in reduced fibroblast and T cell motility, thus helping HIV to overcome T lymphocytes-mediated, chemotaxis-based antiviral response of the host. Also, HIV activates cofilin through chemokine receptor signaling to mediate entry into resting CD4 T cells [83]. These examples together with TBSV-mediated inhibition of cofilin/actin functions suggest that the actin network could be a major target for viruses to facilitate their replication in host cells. Cofilin and actin are also major targets of bacterial pathogens infecting mammalian cells, suggesting that the dynamics of actin network is key component and pathogenicity determinant [84].

### Summary

#### Virus-induced blocking of actin organization facilitates efficient VRC assembly

We show that tombusviruses actively interfere with actin organization during replication. The mechanism of interference is based on interaction of the viral p33 and possibly p92 replication proteins with Cof1p. Since Cof1p is a key actin depolymerization factor [54,55], binding of p33 to Cof1p could inhibit Cof1p interaction with ADP-actin and block actin filament disassembly and recycling of actin monomers to form new actin filaments. Accordingly, co-expression of p33 and p92 in yeast led to large actin-containing areas in the cell, visible under confocal microscope, which is indicative of disorganization of actin patches and lack of dynamic actin function. Based on these observations, we propose that TBSV actively blocks actin organization and remodeling of the actin network via p33-Cof1p interaction and possibly by re-localization of Cof1p from the cytosol to membranes. This block in actin organization is likely temporal, occurring before and during the assembly of the VRC. After VRC assembly, which results in sequestration of newly made p33/p92 molecules into the VRCs, the role of Cof1p in actin organization is likely restored during regular TBSV infections since the actin is required for cell-to-cell movement of plant viruses [85,86].

## Materials and Methods

### Yeast strains and expression plasmids

Expression plasmids, *S*. *cerevisiae* strains, culturing conditions and TBSV repRNA measurements are presented in S1 Materials and Methods.

### Treatment of yeast with Cytochalasin-D

Cytochalasin-D (Santa Cruz Biotechnology) [59] was added to overnight culture of BY4741 (transformed with pHISGBK-CUP1-p33:ADH-DI-72 and pESC-CUP1-His-p92) at a final concentration of 0.03 and 0.06 μg/μl. The yeast was grown for further 24 h at 23°C and total RNA was extracted for Northern blot analysis.

### Treatments of plants and protoplasts with Actin inhibitors

*N*. *benthamiana* leaves were first sap-inoculated with TBSV, and one day later, the same leaves were infiltrated with ethanol or Cytochalasin-D at 80 μg/ml concentration (or as specified in Fig legends) using a syringe to inhibit actin polymerization. Two days later, total RNA was isolated from the inoculated leaves and Northern hybridization was performed to measure tombusvirus RNA accumulation.

In case of inhibitor treatment of the Arp2/3 complex, *N*. *benthamiana* leaves were infiltrated with DMSO (50 μM) or CK-636 (25 or 50 μM) (ApexBio), and 12 h later, the same leaves were inoculated with TBSV-containing sap. One day later the infected leaves were again infiltrated with DMSO (50 μM) or CK-636 (25 or 50 μM). One day later total RNA was isolated and Northern hybridization was performed as described above.

Protoplasts were isolated from *N*. *benthamiana* leaves as previously described [87]. Freshly prepared protoplasts were treated with 70 or 130 μg/ml Cytochalasin-D or 40 μM of Latrunculin-B before electroporation with 1 μg of TBSV RNA. Protoplasts were incubated in the dark for 24 h at room temperature and the total RNA was extracted for Northern hybridization [87].

### Confocal laser microscopic analysis of plant F-actin in the presence of viral proteins

Expression plasmids pGD-T33-RFP or pGD-C36-RFP were transformed into *Agrobacterium* C58C1 separately, and transformed cells were selected on LB plate containing 50 μg/ml kanamycin, 100 μg/ml rifampicin and 5 μg/ml tetracycline. Transformed cells were grown in LB medium containing the antibiotics described above overnight, and suspended in MMA solution (10mM MES pH5.6, 10mM MgCl_2_, 200 μM acetosyringone) at OD_600_ of 1.0 for 3–4 hours. Leaves of 8 weeks old transgenic *Nicotiana benthamiana* plants expressing GFP-mTalin (mouse Talin) [88], which specifically binds to F-actin [70] (a gift from Dr. Michael M. Goodin at University of Kentucky), were infiltrated with *Agrobacterium* in MMA solution. Leaf epidermal cells were observed under confocal laser microscope (Olympus FV1000 microscope) 2 days after infiltration for localization of both GFP-mTalin and p33-RFP/p36-RFP [89]. Note that GFP-mTalin transgenic *Nicotiana benthamiana* plants have normal growth when compared to wild type plants under green house conditions [88]. GFP-mTalin binds to F-actin, and its localization overlaps with phalloidin stained actin filaments [70].

### Super-resolution laser microscopic analysis of yeast actin in the presence of viral proteins

Yeast strain BY4741 was transformed with pESC-T33/DI72 and pYES-T92 [89]. Transformed cells were pre-grown in synthetic complete medium lacking appropriate amino acids and containing 2% glucose overnight at 29°C. Then, yeast cells were grown in synthetic complete medium lacking appropriate amino acids containing 2% galactose at a starting 0.3 OD_600_ for 12–14 hours at 23°C. Cells were harvested, fixed with 3.7% formaldehyde and digested with zymolase 20T to remove cell wall as described in [43]. Treated cells were applied to poly-L-lysine–coated cover slips. The cells on the cover slips were sequentially immersed in methanol for 6 min and acetone for 30s at –20°C. Anti-p33 primary antibodies (a gift from Dr. Herman B. Scholthof, Texas A&M University) were diluted (1:400) in PBS (pH 7.4) containing 0.05% Nonidet P-40 and 1% BSA and incubated overnight with the fixed cells at +4°C. Yeast cells were washed three times with PBS (pH 7.4) with 1% BSA, then incubated subsequently with anti-mouse secondary antibody conjugated to Alexa Flour 647 for 1 h and ATTO488-phalloidin for 1 h in PBS (pH 7.4) with 1% BSA, and washed three times with PBS (pH 7.4) containing 1% BSA for at least 1 h to reduce background. Cells on the cover slip were subjected to super-resolution microscopic observation (N-STORM Super Resolution Microscopy System from Nikon).

### Filipin-based staining of ergoterols in yeasts

To examine the distribution of ergoterol in BY4741 and act1^*ts*^ mutants (act1-121 and act1-132), the yeast strains were co-transformed with plasmids pGBK-Hisp33-CUP1/Gal DI-72 and pGAD-Hisp92-CUP1. Control untransformed and transformed yeasts were grown in SC minimal media supplemented with 2% galactose and 50 μM CuSO_4_ for 24 hours at 23°C, 27°C and 32°C. Cultures were fixed with 3% formaldehyde for 1 h at room temperature. Fixed cells were centrifuged and washed twice with distilled water. Washed cells were incubated with 5 mg/ml filipin complex (Sigma Chemicals) in the dark for 15 min at 23°. Filipin-based fluorescence was observed by spotting 3 μl of the cell suspensions onto poly-lysine microscope slides under UV light microscope using DAPI filter [45].

### Co-purification of Osh6 protein with the p33 replication protein from yeasts

For co-purification of Osh6p protein with the membrane-bound p33 and p92 replication proteins [45], yeast strains BY4741, *cof1-8* ^*ts*^, *cof1-22* ^*ts*^, and the *act1-121* ^*ts*^ mutants were co-transformed with plasmids HpGBK-CUP1-FLAGp33/GAL1-DI-72 and LpGAD-CUP1-FLAGp92 and (or HpGBK-CUP1-Hisp33/Gal1-DI-72 and LpGAD-CUP1-Hisp92 as a control) plus the UpYC-NT plasmids expressing His_6_-tagged Osh6 protein from the *GAL1* promoter (UpYC-NT-Gal1-OSH6). Transformed yeasts cultures were pre-grown in selective SC-ULH^−^ + BCS medium supplemented with 2% glucose for 12 h at 23°C and then transferred to selective medium supplemented with 2% galactose for 24 h at either 23 or 32°C to induce His_6_-Osh6 protein expression from the *GAL1* promoter. Then the cultures were supplemented with 50 μM CuSO_4_ to induce expression of FLAG-p33 and FLAG-p92 or His_6_-p33 and His_6_-p92 from the *CUP1* promoter, and yeast cultures were grown for an additional 4 h at 23°C or 32°C. The cultures were centrifuged washed once with phosphate-buffered saline (PBS), and then incubated in PBS buffer containing 1% formaldehyde for 1 h on ice to cross-link proteins [45]. Finally, yeast cultures were washed in PBS and proteins were FLAG-affinity purified as described previously [45].

## Supporting Information

S1 Materials and Methods Supplementary Materials and Methods. (DOC)

S1 Fig Testing Cof1p mutants for their ability to inhibit TBSV repRNA accumulation in yeast. (A) Accumulation of TBSV repRNA in *cof1-5* ^*ts*^ or wt yeast at the semi-permissive (32°C) temperature. To launch TBSV repRNA replication, we expressed His_6_-p33 and His_6_-p92 from the copper-inducible *CUP1* promoter and TBSV DI-72(+) repRNA from the constitutive *ADH1* promoter in the parental (BY4741) and *cof1-5* ^*ts*^ yeast strains. Northern blot analysis was used to detect DI-72(+) repRNA accumulation, which was normalized based on 5S rRNA. Each experiment was repeated three times. (B) Testing Cof1p mutants defective in Act1p binding for their ability to inhibit TBSV repRNA accumulation in yeast. (C) Testing Cof1p mutants that bind Act1p for their ability to inhibit TBSV repRNA accumulation in yeast. See further details in panel A. (D) Cof1p mutant proteins interact with the TBSV p33 replication protein in yeast. The split ubiquitin assay was used to test binding between p33 and Cof1p in yeast. The bait p33 was co-expressed with the shown prey proteins. *SSA1* and the empty prey vector (NubG-X) were used as positive and negative controls, respectively. The experiment with wt Cof1p is encircled.
(EPS)

S2 Fig Over-expression of the native Cof1p inhibits TBSV repRNA accumulation in yeast. (A-B) The wt or *cof1-8* ^*ts*^ yeast co-expressed the His_6_-p33 and His_6_-p92 from the copper-inducible *CUP1* promoter and TBSV DI-72(+) repRNA from the constitutive *ADH1* promoter. Over-expression of the untagged native Cof1p was done from the *GAL1* promoter. Top images: Northern blot analysis of TBSV repRNA in yeast samples over-expressing Cof1p or without over-expression is shown. repRNA replication took place for 24 hours at 23°C or 32°C in wt or *cof1-8* ^*ts*^ yeast before RNA analysis. The accumulation level of DI-72(+) repRNA (shown in percentage) was normalized based on 18S rRNA.
(EPS)

S3 Fig Confocal laser microscopic images of TBSV-infected plant cells expressing TBSV p33 replication protein. The GFP-mTalin transgenic *N*. *benthamiana* plants were agro-infiltrated with a plasmid expressing p33-RFP and infected with TBSV using sap inoculation. Note the localization of large p33-RFP containing areas (i.e., replication organelles) frequently at the intersection of actin cables. The bars represent 20 μm.
(TIF)

S4 Fig Confocal laser microscopic images of CIRV-infected plant cells expressing CIRV p36 replication protein. The GFP-mTalin transgenic *N*. *benthamiana* plants were agro-infiltrated with a plasmid expressing p36-RFP and infected with CIRV using sap inoculation. Note the localization of large p36-RFP containing areas frequently at the intersection of actin cables. The bars represent 20 μm.
(TIF)

S5 Fig Confocal laser microscopic images of uninfected plant cells. The GFP-mTalin transgenic *N*. *benthamiana* plants were agro-infiltrated with an empty pGD plasmid. The bars represent 20 μm.
(TIF)

S6 Fig Enhanced co-purification of Osh6p with p33/p92 replication proteins from actin mutant yeast. After cross-linking, the FLAG-tagged p33 and FLAG-p92 were purified from solubilized membranous fraction of yeast extracts using a FLAG-affinity column. Top panel: Western blot analysis of the co-purified 6xHis-tagged Osh6p with anti-His antibody in the affinity-purified preparations. Middle panel: Western-blot analysis of the same samples as in the top panel, but using anti-FLAG antibody. Bottom panels: Western blot analysis of 6xHis-Osh6p with anti-His antibody in the total protein extract from yeast expressing the shown proteins. CB: Coomassie-stained SDS-PAGE of total protein extract. Each experiment was repeated two times.
(EPS)

S1 Video 3D Super Resolution laser microscopic image of a yeast cell. The yeast cell replicating TBSV repRNA was imaged to detect the p33 replication protein via Alexa Flour 647 and actin filaments through staining with ATTO488-phalloidin. The bars represent 1 μm. The boxed area represents the 3D image to visualize the localization of actin and p33 replication protein in yeast. The image was prepared by a Nikon Super Resolution Microscope N-STORM and image processing was performed using NIS-element software.
(AVI)
