# Supplementary material for: Viral Replication Protein Inhibits Cellular Cofilin Actin Depolymerization Factor to Regulate the Actin Network and Promote Viral Replicase Assembly
Source: PLoS Pathog. 2016 Feb 10;12(2):e1005440. doi: 10.1371/journal.ppat.1005440 (PMC4749184; doi:10.1371/journal.ppat.1005440)
Supplement: S1 Materials and Methods — (DOC) [file ppat.1005440.s001.doc]

**SUPPLEMENT:**

**Supplementary Materials and Methods:**

**Yeast strains and expression plasmids:** *S. cerevisiae* strains BY4741 (*MATa his3Δ1 leu2Δ0 met15Δ0 ura3Δ0*) was obtained from Open Biosystems (Huntsville, AL, USA). The temperature sensitive mutant *cof1-8* from the library of temperature-sensitive (ts) mutants of yeast was kindly provided by [Charles Boone](http://www.ncbi.nlm.nih.gov/pubmed/?term=Boone C%5Bauth%5D) (University of Toronto) [1]. Temperature-sensitive yeast strains were transformed and cultured as described earlier [2]. The COF1 mutant strains (with Leucine selection marker) were kindly provided by Dr. David Drubin [3]. The GFP-cofilin construct (UpRS316-COF1 with internal GFP), which was provided by Dr. David Drubin, has S65T-GFP and a 12–amino acid linker inserted in between amino acids N74 and G75 of Cof1p [4].

The temperature sensitive cofilin and actin mutants [1] were used to induce TBSV repRNA replication by adding 50 μM CuSO_4_ to the growth medium as described earlier [5,6]. The pHISGBK-CUP1-p33/ADH1-DI-72 (which carries p33 under *CUP1* promoter and DI-72 repRNA under *ADH1* promoter) has been described earlier [5]. As *COF1* mutant yeast strains [3] were transformed with plasmid pESC-CUP1-His-p92 (URA3), which was generated with *URA3* selection marker, by transferring the CUP-p92 cassette from pGAD-His-p92 [6].

The yeast strain carrying the *COF1* gene under control from the *TET* promoter was obtained from Open Biosystems. After the appropriate yeast growth, 10 mg/L of doxycycline was added to down-regulate *COF1* gene expression as described earlier [7,8]. For the Cof1p over-expression experiments, we used the zz- (a truncated Protein A-based) and His_6_-tagged *COF1* (Open Biosystems), which is expressed from the *GAL1* promoter. The over-expression of Cof1p was induced by growing the yeast on medium containing 2% galactose.

For the over-expression of untagged, native Cof1p, the *COF1* gene was amplified from a yeast genomic DNA preparation by primers #4754 (CGCGGATCCATGTCTAGATCTGGGTATGCTAAATTT) and #4755 (CGGCTCGAGTTAATGAGAACCAGCGCCTCTGCTGACTC). The amplified PCR product contained a stop codon at the 3’ end of the *COF1* ORF. The PCR product was digested with *Bam*HI and *Xho*I enzymes and cloned into pYES plasmid resulting into pYES-COF1STOP plasmid.

**Construction of pGD-T33-RFP, and pGD-C36-RFP.** To generate pGD-2x35S-L, sequence of 35S promoter followed by the tobacco etch virus non-translated leader sequence (L) was amplified from pGD-L [9] with primers pairs #6074 (GGAAGATCTCATGGTGGAGCACGACACTCTCG) /#2916 (GCCGGATCCGGCTATCGTTCGTAAATGGTG), digested with *Bgl*II/*BamH*I and inserted into *BamH*I digested pGD [10], resulting pGD-2x35S-L. To generate C-terminal Flag-tagged vector pGD-CFlag, sequence of multi-cloning sites with Flag-tag was amplified from pRS315-CFlag [11] with primer pair #6072 (CCGTGATCATCTAGAACTAGTGGATCCCCCG)/ #6073 (ACGCGTCGACTTACTCGAGGCCAGATCTACCAC), digested with *Bcl*I/*Sal*I and inserted into *BamH*I/*Sal*I digested pGD-2x35S-L. To generate binary vector expressing TBSV p33 or CIRV p36, which were fused with RFP at the C-terminus, firstly mTagRFP sequence was amplified with #5649 (GGAAGATCTATGGTGTCTAAGGGCGAAGAG) and #5652 (CCGCTCGAGTTAATTAAGTTTGTGCCCCAGTTTGC) from pSITEII-6C1 [12], digested with *Bgl*II/*Xho*I, and inserted into *Bgl*II/*Xho*I-digested pGD-CFlag, generating pGD-CRFP. p33 or p36 ORF sequence was PCR-amplified from pESC-T33/DI72 [13] or pESC-C36/DI1 [14] using primer pair #6075 (CGCGGATCCTAAACAATGGAGACCATCAAGAGAATG) /#5837 (GCCCTGCAGTTTGACACCCAGGGACTCCTG) or #6184 (CGCGGATCCTAAACAATGGAGGGTTTGAAGGCTG) /#6185 (GGCATGCATTTTGACACCGAGGGATTCC), then digested with *BamH*I/*Pst*I or *BamH*I/*Nsi*I respectively, and separately inserted into pGD-CRFP digested with *BamH*I/*Pst*I, generating binary vectors pGD-T33-RFP or pGD-C36-RFP, expressing p33-RFP or p36-RFP.

**Yeast growth conditions and measurements of viral RNA replication and viral protein levels.** For the induction of TBSV repRNA replication, 50 μM CuSO_4_ was added to ULH^-^ medium whenever *cof1^Leu^* mutants were used [3]. However, for *cof1-8* and actin mutants, which were transformed with pHISGBK-CUP1-p33:ADH-DI-72, LpGAD-Hisp92-CUP1 and pYES-NT vectors or ZZ-tagged COF1 vector. For over expression studies, the yeast was pre-grown with ULH^-^ glucose (2%) medium to 0.5 OD_600_. Later, the yeast cultures were centrifuged and the pellet was washed with ULH^-^ galactose (2%) medium. Finally, ULH^-^ galactose (2%) medium containing 50 μM CuSO_4_ to induce viral replication and Cof1p over-expression for 36 h. For temperature sensitive mutant yeasts, the cultures were grown either at permissive (23 ºC) or semi-permissive (32ºC) temperature for partial inactivation of Cof1p or Act1p. Total RNA and protein analysis in Northern and Western blots was conducted as described earlier [15,16].

Yeast temperature sensitive Act1 and Arp2 strains and BY4741 as control, were co-transformed with HpGBK-CUP1-Hisp33/ADH-DI-72 and LpGAD-Hisp92-CUP1 [17], using a tube format. The transformed yeast strains were grown at 23°C in SC-HL^−^ (synthetic complete media without histidine and leucine) medium with 2% glucose + BCS for 12 h at 23°C. Then, the liquid culture from each tube was divided into two separate culture tubes, centrifuged; and the liquid was replaced with SC-HL^−^ medium with 2% glucose containing 50 μM CuSO_4_. The yeast cultures were grown for additional 24 hours at either 23°C (permissive temperature) or 27/32°C (semi-permissive temperature depending on the nature of temperature-sensitive mutant) before sample collection for total RNA extraction and Northern analyses.

**In vitro TBSV replication assay.** For the in vitro TBSV replication assay, the *cof1-8^ts^* and WT (BY4741) yeast strains were transformed with pHISGBK-CUP1-p33 and pESC-CUP1-His-p92. The selected transformants were grown at 23 ºC until reaching 1.0 OD_600_. Then, the cultures were diluted to 0.2 OD_600_ and grown until reaching 1.0 OD_600_ at semi-permissive temperature (32 ºC). To express p33 and p92 in yeast, CuSO_4_ was added for 30 min before harvesting of yeast cultures. ~100 mg of yeast pellet was lysed in 600 μl of yeast lysis buffer as explained in [6,18,19]. By using T7 RNA polymerase in vitro RNA was transcribed from DI-72 template. ~500 ng of purified DI-72 RNA was mixed with yeast cell free extract and the in vitro replication reaction was conducted at 25ºC as explained earlier [6,18,19].

Nt-ADF2 clone was kindly provided by Dr. Alice Cheung (University of Massachusetts). The primers #4774 (CCGAGATCTatggcgaatgctgcgtctggaatggctgtgctagac) and #4775 (CGCCTCGAGtcaaagtgctcgtgactttataatgtcaaagctca) were used for PCR to obtain pGEX-NT-ADF2 at *Bam*HI and *Xho*I sites in pGEX-NSRT (modified pGEX2-T). The pGEX-NT-ADF2, MBP tagged p33 and MBP tagged p92 [6,20] were separately transformed into *E. coli* (BL21, Codon Plus) strain and tagged protein transcription was induced by adding IPTG to the bacterial growth medium [6,20]. The proteins were affinity purified and added into in vitro reaction (p33=400ng, p92= 100ng and NT-ADF2 1X=200ng). The synthesized RNAs from the CFE-based assays were analyzed on 8% PAGE denaturing gel [21,22].

**TBSV replication in yeast cell free extracts**. Cell-free extracts (CFE) from yeast strains BY4741, cof1-8 and act 1-132 were prepared as described previously [21]. The in vitro CFE assays were carried out with 0.1 μg of MBP-tagged p33 and MBP-p92 (affinity-purified from *E. coli*), 0.5 μg of in vitro-transcribed DI-72 (+)repRNA, and 2 μl of CFE in a 20-μl final volume. The mixtures were incubated at 25°C for 3 h, and the amount of newly synthesized ^32^P-labeled repRNA was analyzed in denaturing polyacrylamide/urea gels as described previously [21].

For the mix and match CFE fractionation-based assay, the CFEs were prepared from yeast strains BY4741, cof1-8 and act1-132 and then were subjected to centrifugation at 35,000 g for 30 min to separate membrane and soluble fractions. These separated fractions were mixed in various combinations as described in the figure legend.

For the CFE assay based on pre-expressed p33 and p92 in yeast, the yeast strains BY4741 cof1-8, and act1-132 were co-transformed with plasmids HpGBK-CUP1-Hisp33 and LpGAD-CUP1-Hisp92. Transformed yeasts were grown and CFEs were prepared as described [21]. Replicase reactions were carried out with 2 μl yeast CFE with pre-expressed p33 and p92, and 500 ng DI-72 (+)repRNA in a 20-μl final volume. The mixtures were incubated at 25°C for 3 h and the amount of newly synthesized ^32^P-labeled repRNA was analyzed in denaturing polyacrylamide/urea gels as described previously [21].

**In vitro replicase assay using yeast membrane-enriched fractions.** The BY4741 or *act1^ts^* mutant strains carrying plasmids HpGBK-CUP1-Hisp33/Adh-DI-72 and LpGAD-CUP1-Hisp92 were pre-grown in SC media supplemented with 2% glucose at 23 °C for 12 h. Then, the yeast cultures were transferred to SC media supplemented with 2% glucose and 50 μM CuSO_4_ and incubated at 23°C, 27 ºC or 32°C for 24 h. Yeast cultures were collected by centrifugation and cells were broken with glass beads in a Genogrinder to obtain membrane-enriched fractions containing the active TBSV replication complexes including the repRNA as described [9]. Comparable amounts of replicase preparations were used (based on p33 levels) to perform the in vitro assays in 100 μl containing 25 μl of the normalized membrane-enriched fractions, 50 mM Tris–Cl pH 8.0, 10 mM MgCl_2_, 10 mM DTT, 0.2 μl RNase inhibitor, 1 mM ATP, 1 mM CTP, 1 mM GTP and 0.1 μl of *α*^32^P UTP (3000 Ci/mmol). Reaction mixtures were incubated for 2 h at 25 °C and then the RNA were obtained by phenol/chloroform extraction and isopropanol/ammonium acetate (10:1) precipitation. The α^32^P UTP-labeled repRNA products were separated on 8% acrylamide/8M urea gels and analyzed using a phosphorimager.

**Actin cytoskeleton staining by Rhodamine-Phalloidin in yeast.** Rhodamine-Phalloidin (Invitrogen) staining was used to visualize actin organization in yeast cultures. Staining was performed by the standard procedure of yeast cells fixation, followed by staining [23,24]. The BY4741 yeast strain was transformed with pHISGBK-CUP1-p33/ADH-DI-72 alone, pESC-CUP1-His-p92 alone or together with the two plasmids. The untransformed *cof1-8^ts^* and BY4741 along with transformed BY4741 were grown at 30 ºC till 1.0 OD_600_. The cells were harvested and washed with phosphate-buffered saline (PBS, 50 mM Potassium phosphate pH7.4, 50 mM NaCl). Then, yeast cells were fixed by fixative solution (3.7% paraformaldehyde in PBS, pH 7.0) for 30 min at room temperature. Cells were washed with PBS buffer and 1% BSA (bovine serum albumin) for 30 sec. 5 units of Rhodamine-Phalloidin (Invitrogen) were added to 200 μl of BSA buffer and added to each culture. Cells were again washed three times with PBS and 50 μl of mounting medium (50% glycerol in PBS and 1 mg/ml of *p-*phenylene diamine) was added for each culture. Cells were visualized by using rhodamine filter in a confocal laser microscope (Olympus FV1000).

**VIGS to knock down *ADF2* level in *Nicotiana benthamiana***. TRV based VIGS vectors (TRV1 and TRV2) [25] were used for silencing of actin depolymerizing factor *NbADF2* gene [26]. The primers #4770 (CCGGGATCCgcaagctgaagtttctggaattgaaagctaagagg) and #4771 (CGCCTCGAGcaaggattgctaattgttaccagctccaaaccaaag) were used to PCR-amplify *NbADF2* gene from *N. benthamiana* DNA. The PCR products were digested with *Bam*HI/*Xho*I and were ligated into TRV2 VIGS vector to generate pTRV2-NB-ADF. The vectors (pTRV1, pTRV2-NB-ADF and pTRV2-PDS) were transformed into an *Agrobacterium* strain (GV3101) and infiltrated into 3 weeks old *N. benthamiana* plants as described [27]. 10 days after agro-infiltration, TBSV virion preparation was sap inoculated using upper leaves. At 4 day postinoculation (dpi), the systemically infected leaves were collected and total RNA was extracted as described earlier [28,29]. TBSV RNA accumulation was measured by using Northern blot and TBSV specific probes as described earlier [29-31].

**Construction of plant myosin motor dominant negative mutants.** To generate N-terminal Flag-tagged *Nicotiana benthamiana* myosin dominant negative expression construct, we PCR-amplified the multi-cloning sites with Flag tag from pRS315-NFlag [14] by primer pairs # 6070 (CCGTGATCATAAACAATGGCTAGATCTGGCACTAGTGACTACAAGG) and # 6071 (ACGCGTCGACGGTATCGATAAGCTTGATATC), followed by digestion of the PCR product with BclI/SalI and insertion into BamHI/SalI digested pGD-2x35S-L, resulting pGD-NFlag. The *Nicotiana benthamiana* Myosin VIII-1 or XI-K tail sequence [32] were PCR-amplified from *Nicotiana benthamiana* cDNA using primer pair #6651 (CGCGGATCCCGGACTCTCCATGGCATTTTACGTGTGC) and #6652 (ACGCGTCGACTTAGTTAAACCTTGTGCTATTCCTCCTTCCCC) or #6650 (CGCGGATCCGTACTTGGAAATGCAGCAAAAATTATTCAAAGAC) and #6450 (CGCGGATCCTCACCAACTGGAAAAACTTTATCTGCACGGCC), respectively. The PCR products were digested with BamHI/SalI, and inserted into BamHI/SalI digested pGD-NFlag, resulting in construct pGD-Flag-NbMyo-VIII-1-DN and pGD-Flag-NbMyo-XI-K-DN, respectively.

**Expression of Myosin tail sequences in *Nicotiana benthamiana* leaves.** Expression plasmids pGD-Flag-NbMyo-VIII-1-DN, pGD-Flag-NbMyo-XI-K-DN containing Myosin tail domain, or pGD-p19 containing TBSV-encoded suppressor of RNA silencing were transformed into *Agrobacterium* C58C1 respectively. Transformed *Agrobacterium* cells were grown in LB medium containing 50 μg/ml kanamycin, 100 μg/ml rifampicin and 5 μg/ml tetracycline overnight, and suspended in MMA solution (10mM MES pH5.6, 10mM MgCl_2_, 200uM acetosyringone) at OD_600_ of 1.5 for 3-4 hours. *Agrobacterium* carrying p19 expression plasmids (0.3 OD_600_) was mixed with *Agrobacterium* carrying either pGD-Flag-NbMyo-VIII-1-DN or pGD-Flag-NbMyo-XI-K-DN (1.2 OD_600_) before infiltration. Plasmid expressing a red fluorescent protein (RFP) was used as a control. 1day after agroinfiltration, CNV replication was launched via agroinfiltration of the same leaves. Samples from the dual agroinfiltrated leaves were collected 3 days after initiation of CNV replication, and subjected for Northern blot analysis.

**Confocal laser microscopy.** The BY4741 and *cof1-8^ts^* mutant yeast strains were transformed with CFP-tagged Pex13 (pGAD-ADH1-PEX13-CFP, Leu^-^) and YFP-tagged p33 (pGAD-ADH1-YFP-p33, His^-^) [33]. The selected transformants were grown at 28 ºC for 24 h and were analyzed by confocal laser microscopy as described earlier [34,35].

**Split-Ubiquitin assay in yeast with *cof1* mutants.** For split-ubiquitin assay, *COF1* mutants were PCR-amplified from the genomic DNA of the mutant yeast strains by using primers #2988 (GCCGGTACCATGTCTAGATCTGGTGTTGC) and #2989 (CGGCTCGAGTTAGCTAGCATGAGAACCAGCGCCTC). The PCR-amplified products were digested with *Bam*HI and *Xho*I to clone into pPRN plamid (Dual Systems) to yield pPRN-COF1-5, pPRN-COF1-8, pPRN-COF1-10, pPRN-COF1-20, pPRN-COF1-21 and pPRN-COF1. The bait plasmids, pGAD-BT3-N-His33 and negative control pPR-N-RE (NubG) vectors are explained in [36,37]. The prey mutant plasmids and bait p33 plasmids were co-transformed in NMY51 yeast strain and final selection of split-ubiquitin was confirmed on TLHA^−^ (Trp^−^/Leu^−^/His^−^/Ade^−^) selection at 28 ºC as described earlier [5,37].

**Co-purification of cellular proteins with p33 from yeast membranes.** Various yeast strains, such as cof1-8, act-132-ts and BY4741, were transformed with plasmids pGBK-HIS-CUP1-Flag33/GAL1-DI-72 [38] pGAD-CUP1-Flag92 [39] and the 6xHis-tagged cellular proteins, such as pYC(Ura)-Gal-DED1 [40] or pYES(Ura)-Gal-VAP27-1. The transformed yeast cells were pre-grown in SC-ULH^−^ media supplemented with 2% glucose and 100 μM BCS at 23°C. For protein purification, yeast cells were centrifuged at 2,000 rpm for 3 min, washed with SC-ULH^−^ media containing 2% galactose, followed by re-suspension in SC-ULH^−^ media supplemented with 2% galactose and 100 μM BCS. Following 24 h of culturing at 27°C, the media was changed to ULH^−^ media supplemented with 2% Galactose and 50 µM CuSO_4_. The protein induction lasted for 6 h at 27°C, then yeasts were pelleted and the membrane-bound replicase was solibilized and FLAG-affinity-purified according to a previously described [41]. Total protein fraction (after breaking cells and balanced based on total proteins amount) and the purified fraction, eluted from anti-FLAG M2-agarose affinity resin column (balanced based on Flag-p33 amount) were analyzed to measure the co-purified 6xHis-tagged cellular proteins. The purified Flag-p33 and Flag-p92 were detected by Western blot using anti-Flag antibody, followed by anti-mouse antibody conjugated to alkaline phosphatase. Colorimetric detection was performed with NBT and BCIP.

**Figure legends**

**Supplementary materials:**

**Supplementary figure legend:**

**S1 Figure.** Testing Cof1p mutants for their ability to inhibit TBSV repRNA accumulation in yeast. (A) Accumulation of TBSV repRNA in *cof1-5^ts^* or wt yeast at the semi-permissive (32ºC) temperature. To launch TBSV repRNA replication, we expressed His_6_-p33 and His_6_-p92 from the copper-inducible *CUP1* promoter and TBSV DI-72(+) repRNA from the constitutive *ADH1* promoter in the parental (BY4741) and *cof1-5^ts^* yeast strains. Northern blot analysis was used to detect DI-72(+) repRNA accumulation, which was normalized based on 5S rRNA. Each experiment was repeated three times. (B) Testing Cof1p mutants defective in Act1p binding for their ability to inhibit TBSV repRNA accumulation in yeast. (C) Testing Cof1p mutants that bind Act1p for their ability to inhibit TBSV repRNA accumulation in yeast. See further details in panel A. (D) Cof1p mutant proteins interact with the TBSV p33 replication protein in yeast. The split ubiquitin assay was used to test binding between p33 and Cof1p in yeast. The bait p33 was co-expressed with the shown prey proteins. *SSA1* and the empty prey vector (NubG-X) were used as positive and negative controls, respectively. The experiment with wt Cof1p is encircled.

**S2 Figure.** Over-expression of the native Cof1p inhibits TBSV repRNA accumulation in yeast. (A-B) The wt or *cof1-8^ts^* yeast co-expressed the His_6_-p33 and His_6_-p92 from the copper-inducible *CUP1* promoter and TBSV DI-72(+) repRNA from the constitutive *ADH1* promoter. Over-expression of the untagged native Cof1p was done from the *GAL1* promoter. Top images: Northern blot analysis of TBSV repRNA in yeast samples over-expressing Cof1p or without over-expression is shown. repRNA replication took place for 24 hours at 23 ºC or 32 ºC in wt or *cof1-8^ts^* yeast before RNA analysis. The accumulation level of DI-72(+) repRNA (shown in percentage) was normalized based on 18S rRNA.

**S3 Figure.** Confocal laser microscopic images of TBSV-infected plant cells expressing TBSV p33 replication protein. The GFP-mTalin transgenic *N. benthamiana* plants were agro-infiltrated with a plasmid expressing p33-RFP and infected with TBSV using sap inoculation. Note the localization of large p33-RFP containing areas (i.e., replication organelles) frequently at the intersection of actin cables. The bars represent 20 μm.

**S4 Figure.** Confocal laser microscopic images of CIRV-infected plant cells expressing CIRV p36 replication protein. The GFP-mTalin transgenic *N. benthamiana* plants were agro-infiltrated with a plasmid expressing p36-RFP and infected with CIRV using sap inoculation. Note the localization of large p36-RFP containing areas frequently at the intersection of actin cables. The bars represent 20 μm.

**S5 Figure.** Confocal laser microscopic images of uninfected plant cells. The GFP-mTalin transgenic *N. benthamiana* plants were agro-infiltrated with an empty pGD plasmid. The bars represent 20 μm.

**S6 Figure.** Enhanced co-purification of Osh6p with p33/p92 replication proteins from actin mutant yeast. After cross-linking, the FLAG-tagged p33 and FLAG-p92 were purified from solubilized membranous fraction of yeast extracts using a FLAG-affinity column. Top panel: Western blot analysis of the co-purified 6xHis-tagged Osh6p with anti-His antibody in the affinity-purified preparations. Middle panel: Western-blot analysis of the same samples as in the top panel, but using anti-FLAG antibody. Bottom panels: Western blot analysis of 6xHis-Osh6p with anti-His antibody in the total protein extract from yeast expressing the shown proteins. CB: Coomassie-stained SDS-PAGE of total protein extract. Each experiment was repeated two times.

**S1 Video Figure.** 3D Super Resolution laser microscopic image of a yeast cell. The yeast cell replicating TBSV repRNA was imaged to detect the p33 replication protein via Alexa Flour 647 and actin filaments through staining with ATTO488-phalloidin. The bars represent 1 μm. The boxed area represents the 3D image to visualize the localization of actin and p33 replication protein in yeast. The image was prepared by a Nikon Super Resolution Microscope N-STORM and image processing was performed using NIS-element software.

**REFERENCES:**

1. Li Z, Vizeacoumar FJ, Bahr S, Li J, Warringer J, et al. (2011) Systematic exploration of essential yeast gene function with temperature-sensitive mutants. Nat Biotechnol 29: 361-367.

2. Shah Nawaz-Ul-Rehman M, Reddisiva Prasanth K, Baker J, Nagy PD (2013) Yeast screens for host factors in positive-strand RNA virus replication based on a library of temperature-sensitive mutants. Methods 59: 207-216.

3. Lappalainen P, Fedorov EV, Fedorov AA, Almo SC, Drubin DG (1997) Essential functions and actin-binding surfaces of yeast cofilin revealed by systematic mutagenesis. EMBO J 16: 5520-5530.

4. Okreglak V, Drubin DG (2007) Cofilin recruitment and function during actin-mediated endocytosis dictated by actin nucleotide state. J Cell Biol 178: 1251-1264.

5. Mendu V, Chiu M, Barajas D, Li Z, Nagy PD (2010) Cpr1 cyclophilin and Ess1 parvulin prolyl isomerases interact with the tombusvirus replication protein and inhibit viral replication in yeast model host. Virology 406: 342-351.

6. Panaviene Z, Panavas T, Serva S, Nagy PD (2004) Purification of the Cucumber necrosis virus replicase from yeast cells: Role of coexpressed viral RNA in stimulation of replicase activity. J Virol 78: 8254-8263.

7. Barajas D, Li Z, Nagy PD (2009) The Nedd4-Type Rsp5p Ubiquitin Ligase Inhibits Tombusvirus Replication by Regulating Degradation of the p92 Replication Protein and Decreasing the Activity of the Tombusvirus Replicase. J Virol 83: 11751-11764.

8. Serviene E, Jiang Y, Cheng C-P, Baker J, Nagy PD (2006) Screening of the Yeast yTHC Collection Identifies Essential Host Factors Affecting Tombusvirus RNA Recombination. J Virol 80: 1231-1241.

9. Barajas D, Jiang Y, Nagy PD (2009) A unique role for the host ESCRT proteins in replication of Tomato bushy stunt virus. PLoS Pathog 5: e1000705.

10. Goodin MM, Dietzgen RG, Schichnes D, Ruzin S, Jackson AO (2002) pGD vectors: versatile tools for the expression of green and red fluorescent protein fusions in agroinfiltrated plant leaves. Plant J 31: 375-383.

11. Xu K, Nagy PD (2015) RNA virus replication depends on enrichment of phosphatidylethanolamine at replication sites in subcellular membranes. Proc Natl Acad Sci U S A 112: E1782-1791.

12. Martin K, Kopperud K, Chakrabarty R, Banerjee R, Brooks R, et al. (2009) Transient expression in Nicotiana benthamiana fluorescent marker lines provides enhanced definition of protein localization, movement and interactions in planta. Plant J 59: 150-162.

13. Xu K, Huang TS, Nagy PD (2012) Authentic in vitro replication of two tombusviruses in isolated mitochondrial and endoplasmic reticulum membranes. J Virol 86: 12779-12794.

14. Xu K, Lin JY, Nagy PD (2014) The hop-like stress-induced protein 1 cochaperone is a novel cell-intrinsic restriction factor for mitochondrial tombusvirus replication. J Virol 88: 9361-9378.

15. Cheng C-P, Panavas T, Luo G, Nagy PD (2005) Heterologous RNA replication enhancer stimulates in vitro RNA synthesis and template-switching by the carmovirus, but not by the tombusvirus, RNA-dependent RNA polymerase: Implication for modular evolution of RNA viruses. Virology 341: 107-121.

16. Panavas T, Serviene E, Brasher J, Nagy PD (2005) Yeast genome-wide screen reveals dissimilar sets of host genes affecting replication of RNA viruses. PNAS 102: 7326-7331.

17. Li Z, Barajas D, Panavas T, Herbst DA, Nagy PD (2008) Cdc34p ubiquitin-conjugating enzyme is a component of the tombusvirus replicase complex and ubiquitinates p33 replication protein. J Virol 82: 6911-6926.

18. Pogany J, Stork J, Li Z, Nagy PD (2008) In vitro assembly of the Tomato bushy stunt virus replicase requires the host Heat shock protein 70. Proceedings of the National Academy of Sciences 105: 19956-19961.

19. Wang RY-L, Nagy PD (2008) Tomato bushy stunt virus Co-Opts the RNA-binding function of a host metabolic enzyme for viral genomic RNA synthesis. Cell Host Microbe 3: 178-187.

20. Li Z, Pogany J, Tupman S, Esposito AM, Kinzy TG, et al. (2010) Translation elongation factor 1A facilitates the assembly of the Tombusvirus replicase and stimulates minus-strand synthesis. PLoS Pathog 6: e1001175.

21. Pogany J, Stork J, Li Z, Nagy PD (2008) In vitro assembly of the Tomato bushy stunt virus replicase requires the host Heat shock protein 70. Proc Natl Acad Sci U S A 105: 19956-19961.

22. Pogany J, Nagy PD (2008) Authentic replication and recombination of Tomato bushy stunt virus RNA in a cell-free extract from yeast. J Virol 82: 5967-5980.

23. Clark MG, Amberg DC (2007) Biochemical and Genetic Analyses Provide Insight Into the Structural and Mechanistic Properties of Actin Filament Disassembly by the Aip1p‚ÄìCofilin Complex in Saccharomyces cerevisiae. Genetics 176: 1527-1539.

24. David A, Burke D, Strathern JN (2005) Methods in Yeast Genetics: A Cold spring Harbor Laboratory Course Manual. : Cold Spring Harbor Laboratory Press, Cold Spring Harbor, NY.

25. Dinesh-Kumar SP, Anandalakshmi R, Marathe R, Schiff M, Liu Y (2003) Virus-induced gene silencing. Methods in molecular biology (Clifton, NJ) 236: 287-294.

26. Cheng CP, Pogany J, Nagy PD (2002) Mechanism of DI RNA formation in tombusviruses: dissecting the requirement for primer extension by the tombusvirus RNA dependent RNA polymerase in vitro. Virology 304: 460-473.

27. Jaag HM, Nagy PD (2009) Silencing of Nicotiana benthamiana Xrn4p exoribonuclease promotes tombusvirus RNA accumulation and recombination. Virology 386: 344-352.

28. Jaag HM, Pogany J, Nagy PD (2010) A host Ca2+/Mn2+ Ion pump is a factor in the emergence of viral RNA recombinants. Cell Host Microbe 7: 74-81.

29. Jaag HM, Nagy PD (2009) Silencing of Nicotiana benthamiana Xrn4p exoribonuclease promotes tombusvirus RNA accumulation and recombination. Virology 386: 344-352.

30. Cheng CP, Jaag HM, Jonczyk M, Serviene E, Nagy PD (2007) Expression of the Arabidopsis Xrn4p 5‚Ä≤-3‚Ä≤ exoribonuclease facilitates degradation of tombusvirus RNA and promotes rapid emergence of viral variants in plants. Virology 368: 238-248.

31. Panavas T, Nagy PD (2003) Yeast as a model host to study replication and recombination of defective interfering RNA of Tomato bushy stunt virus. Virology 314: 315-325.

32. Avisar D, Prokhnevsky AI, Dolja VV (2008) Class VIII myosins are required for plasmodesmatal localization of a closterovirus Hsp70 homolog. J Virol 82: 2836-2843.

33. Wang RYL, Stork J, Nagy PD (2009) A key role for heat shock protein 70 in the localization and insertion of tombusvirus replication proteins to intracellular membranes. Journal of Virology 83: 3276-3287.

34. Panavas T, Hawkins CM, Panaviene Z, Nagy PD (2005) The role of the p33:p33/p92 interaction domain in RNA replication and intracellular localization of p33 and p92 proteins of Cucumber necrosis tombusvirus. Virology 338: 81-95.

35. Jonczyk M, Pathak KB, Sharma M, Nagy PD (2007) Exploiting alternative subcellular location for replication: tombusvirus replication switches to the endoplasmic reticulum in the absence of peroxisomes. Virology 362: 320-330.

36. Kittanakom S, Chuk M, Wong V, Snyder J, Edmonds D, et al. (2009) Analysis of membrane protein complexes using the split-ubiquitin membrane yeast two-hybrid (MYTH) system. Methods in molecular biology (Clifton, NJ) 548: 247-271.

37. Li Z, Barajas D, Panavas T, Herbst DA, Nagy PD (2008) Cdc34p ubiquitin-conjugating enzyme is a component of the tombusvirus replicase complex and ubiquitinates p33 replication protein. Journal of Virology 82: 6911-6926.

38. Kovalev N, Nagy PD (2013) Cyclophilin a binds to the viral RNA and replication proteins, resulting in inhibition of tombusviral replicase assembly. J Virol 87: 13330-13342.

39. Barajas D, Li Z, Nagy PD (2009) The Nedd4-type Rsp5p ubiquitin ligase inhibits tombusvirus replication by regulating degradation of the p92 replication protein and decreasing the activity of the tombusvirus replicase. J Virol 83: 11751-11764.

40. Chuang C, Prasanth KR, Nagy PD (2015) Coordinated Function of Cellular DEAD-Box Helicases in Suppression of Viral RNA Recombination and Maintenance of Viral Genome Integrity. PLoS Pathog 11: e1004680.

41. Kovalev N, Pogany J, Nagy PD (2012) A Co-Opted DEAD-Box RNA Helicase Enhances Tombusvirus Plus-Strand Synthesis. PLoS Pathog 8: e1002537.
